# A dual-index framework for cold atmospheric plasma dosing: A meta-analysis of parametric control from volumetric redox flux to redox homeostatic status

**DOI:** 10.1016/j.redox.2026.104162

**Published:** 2026-04-08

**Authors:** Xiaofeng Dai, Jitian Li, Tanzeela Nawaz, Chaoqun Ding

**Affiliations:** a Department of Geriatric Endocrinology and Metabolism, International Ward 1, The First Affiliated Hospital of Xi'an Jiaotong University, Xi'an, 710061, China; b National Local Joint Engineering Research Center for Precision Surgery & Regenerative Medicine, Shaanxi Provincial Center for Regenerative Medicine and Surgical Engineering, The First Affiliated Hospital of Xi'an Jiaotong University, Xi'an Jiaotong University, Xi'an, 710061, China; c Clinical Medical Center of Tissue Engineering and Regeneration, The Third Affiliated Hospital of Henan Medical University, Henan Medical University, 601 Jinsui Road, Xinxiang, 453000, China

**Keywords:** Cold atmospheric plasma, Plasma dosage, Parametric control, Reactive oxygen and nitrogen species, Volumetric redox flux, Redox homeostatic status, Systematic review

## Abstract

Cold atmospheric plasma (CAP) has emerged as a versatile therapeutic platform with demonstrated efficacy across diverse disease models. Despite significant preclinical progress, clinical translation remains hindered by the absence of standardized dosing protocols and consensus on dosimetric quantification. This systematic review and meta-analysis synthesizes evidence from 2020 to 2025 to establish a parametric framework for CAP dosing grounded in quantitative analysis of dose-dependent biological responses. We first characterize the biphasic, hormetic nature of CAP biological effects, demonstrating that moderate-to-high doses induce pro-oxidant cytotoxicity via oxidative stress-mediated cell death, whereas low-to-moderate doses activate antioxidant defense pathways favoring cytoprotection. Through systematic analysis of 400+ studies meeting inclusion criteria, we quantitatively map five critical control parameters, frequency, flow rate, voltage, processing time, and gas composition, elucidating their individual and synergistic influences on reactive species profiles and therapeutic outcomes. Our meta-analysis reveals distinct parametric distributions across discharge configurations, treatment modalities, and disease applications. Based on this quantitative synthesis, we propose a dual-index integrative framework for CAP dosage: volumetric reactive oxygen and nitrogen species flux as the physical dosimetric index governing parametric control, and redox homeostatic status as the biological dosimetric index reflecting cellular heterogeneity and therapeutic sensitivity. Current limitations arising from device heterogeneity and treatment form diversity are discussed alongside emerging solutions including machine learning-assisted dose prediction and closed-loop control systems.

## Graphical abstract

Cold atmospheric plasma (CAP) elicits a hormetic, biphasic dose-response curve fundamentally governed by the concentration and composition of delivered reactive oxygen and nitrogen species (RONS). While, low-to-moderate RONS fluxes activate adaptive antioxidant pathways, conferring cytoprotection and enhanced stress resistance, moderate-to-high RONS fluxes overwhelm cellular antioxidant capacity, triggering oxidative damage and programmed cell death. To address this dose-dependent duality, we propose a dual-index integrative framework for CAP dosage comprising ‘volumetric RONS flux’ (ΦRONS) as the physical dosimetric index and ‘redox homeostatic status’ as the biological dosimetric index. ΦRONS integrates three determinant factors, interfacial RONS concentration, transport velocity, and treatment area, into a quantifiable, device-agnostic metric; redox homeostatic status captures biological response through endogenous markers that reflect both the pre-treatment redox setpoint and CAP-induced shifts. Together, these twin indices establish a closed-loop adaptive control architecture bridging plasma device operation and clinical efficacy, offering a roadmap toward reproducible, personalized plasma medicine.

**Figure:**
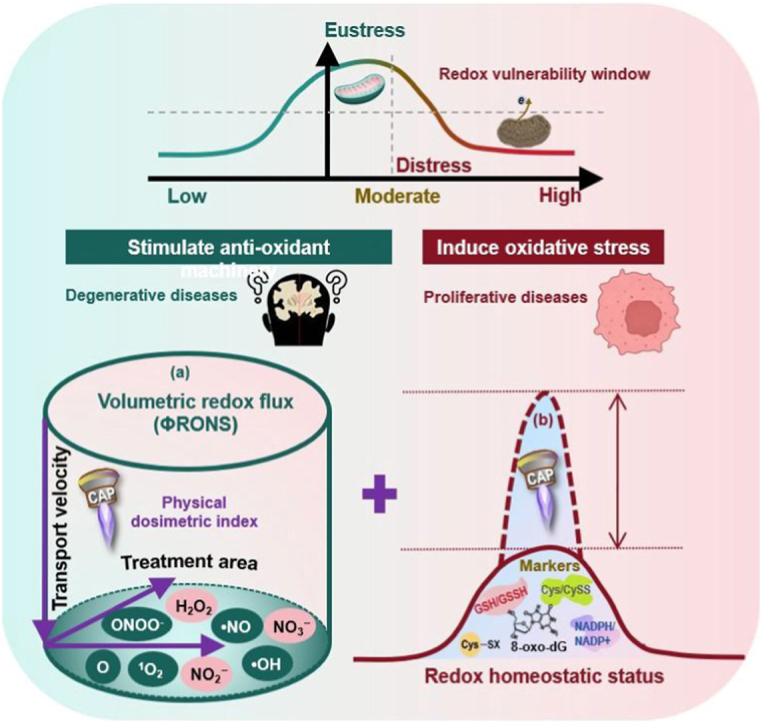

## 1 Introduction

Cold atmospheric plasma (CAP), partially ionized gas generated at near-ambient temperature and atmospheric pressure, has transitioned over the past decade from a physical curiosity to a clinically relevant therapeutic modality [1]. Unlike thermal plasmas that exceed 1000 °C, CAP operates at temperatures close to the room or body temperature, enabling direct application to living tissues without thermal damage [2]. This unique property, combined with its capacity to generate a rich cocktail of reactive oxygen and nitrogen species (RONS) including short-lived radicals like hydroxyl radical (OH·), superoxide anion (·O_2_^−^), singlet oxygen (^1^O_2_), and long-lived species like hydrogen peroxide (H_2_O_2_), ozone (O_3_), nitric oxide (NO), peroxynitrite derivatives (OONO^−^, ONOOH), as well as UV radiation, charged species, and transient electric fields, has positioned CAP as a transformative platform in plasma medicine [[2], [3], [4], [5], [6], [7]].

CAP has exhibited a broad spectrum of biological applications and been canonically used for wound healing [[8], [9], [10], [11], [12], [13], [14], [15]], dental biofilm elimination [16], and corneal infection control [17], largely owing to its efficacy in promoting tissue regeneration and combating infections. Beyond the biomedical field, CAP has been employed in agricultural and environmental contexts [[18], [19], [20]] to enhance biochemical traits and crop sustainability, as reviewed in Ref. [21], with applications ranging from tomato cultivation [22,23] to disinfection of waterborne pathogens [24]. A particularly promising application of CAP is the eradication of antibiotic-resistant bacterial strains, with direct relevance to wound healing and infection control [25,26]. In recent years, considerable CAP related research efforts have been devoted to oncology. The ability of CAP in treating cancers was firstly reported in 2017. Ever since then, incremental evidence has suggested that CAP can effectively target cancer cells while sparing healthy cells [1,27], making it a promising tool for cancer treatment. Building on earlier pioneering in vivo studies demonstrating tumor ablation following plasma delivery [28,29], subsequent research has shown efficacy across a variety of cancer types like melanoma [[30], [31], [32], [33], [34]], glioma [35], glioblastoma [36], head and neck cancer [37], breast cancer [[38], [39], [40], [41], [42], [43]], prostate cancer [44], hepatocellular carcinoma [45], bladder cancer [46], squamous cell carcinoma [47], and pancreatic adenocarcinoma [48,49]. Emerging lines of investigation further extend the therapeutic potential of CAP to chronic inflammatory diseases like atopic dermatitis [[50], [51], [52]] and fibrosis [53], as well as its anti-oxidant potency [54] for, e.g., neuroprotection against excitotoxic injury [55,56].

Yet this very versatility exposes the central paradox confronting plasma medicine, i.e., the same device, operated under seemingly identical conditions, can produce diametrically opposite biological outcomes. CAP has been reported to both induce [[38], [39], [40], [41], [42], [43],^57^] and prevent [55,56] cell demise due to its context- and dose-dependent bidirectional roles in both exacerbating and alleviating cellular oxidative stress [58,59]. This biphasic behavior is not contradictory but rather reflects the fundamental principle of hormesis, where low-dose exposure activates adaptive cellular protection while high-dose exposure triggers death pathways [54,60]. The critical challenge, therefore, is not whether CAP works, but how to deliver the right dose for the right indication with reproducible precision.

Current CAP research suffers from what might be termed ‘dosimetric fragmentation’. Studies from different labs document their results in differed parameter combinations like exposure time, applied voltage, power, energy density, or simply as device-specific operating parameters. This Tower of Babel has prevented cross-study comparison, impeded meta-analysis, and critically delayed regulatory approval and clinical protocol standardization. Thus, heterogeneous device configurations and parameter reporting has become one of the greatest barriers hindering the clinical translation of CAP.

Current CAP research suffers from what might be termed ‘dosimetric fragmentation’. Studies from different labs document their results in differed parameter combinations like exposure time, applied voltage, power, energy density, or simply as device-specific operating parameters. This Tower of Babel has prevented cross-study comparison, impeded meta-analysis, and challenged regulatory approval and clinical protocol standardization. While significant progress has been made, including the publication of the DIN SPEC 91315 standard, which establishes general requirements for plasma sources in medicine [61], and ongoing efforts to harmonize reporting practices across the field [62], heterogeneous device configurations and parameter reporting remain among the greatest barriers hindering the widespread clinical translation of CAP.

The solution lies not in mandating a single universal dosimeter, an approach that ignores the fundamental diversity of CAP devices and applications, but in establishing parametric control frameworks capable of mapping controllable inputs to biological outputs via quantifiable mediating metrics. For instance, recent advances in plasma control engineering, including closed-loop feedback systems and machine learning-assisted parameter optimization, may offer unprecedented opportunities to transform CAP from an empirical tool into a precisely titratable therapeutic [[63], [64], [65], [66]]. Under this context, this review addresses the dosing problem through three interconnected analyses. First, we characterize the dose-dependent biological impact of CAP, examining reported quantitative thresholds, identified in specific plasma device-cell type combinations, for pro-oxidant versus antioxidant effects. Second, we systematically identify and evaluate five critical control parameters, i.e., frequency, flow rate, voltage, processing time, and gas composition, elucidating their individual and interactive effects on RONS production and therapeutic efficacy. Third, we propose a dual-index integrative framework for CAP dosage that couples volumetric redox flux, a physical metric governing parametric control, with redox homeostatic status, a biological metric that reports cellular heterogeneity and responsiveness. We conclude by examining current limitations and future directions, particularly emphasizing challenges imposed by device heterogeneity and treatment form diversity.

## 2 Dose-dependent biological impact

**Fig. 1:**
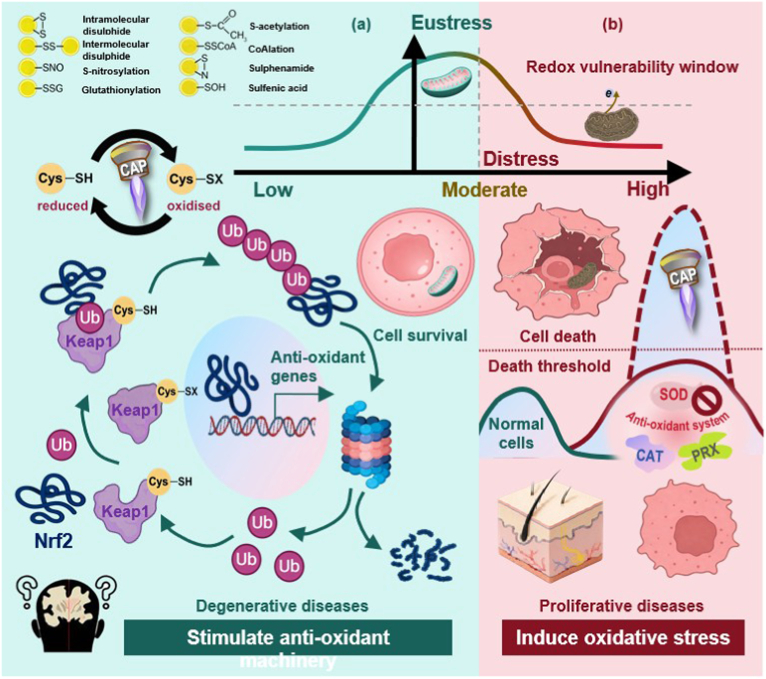
**Dose-dependent biphasic biological effects of cold atmospheric plasma.** Hormetic dose-response curve illustrating the distinct and opposing outcomes elicited by low-to-moderate versus moderate-to-high redox fluxes. CAP elicits a non-monotonic, biphasic biological response fundamentally determined by the concentration and composition of delivered RONS. **(a)** Low-to-moderate doses activate adaptive antioxidant pathways, conferring cytoprotection and enhanced stress resistance. Mechanically, RONS can stimulate the anti-oxidant machinery by inducing reversible oxidation of specific cysteine residues (Cys151, Cys273, Cys288) on KEAP1, leading to the liberation of Nrf2 from KEAP1-mediated ubiquitination. Stabilized Nrf2 translocates to the nucleus, and binds ARE in the promoter regions of anti-oxidant genes like GPX4 and GSH. This biological impact enables the application of CAP in the treatment of degenerative syndromes like Alzheimer's disease. **(b)** Moderate-to-high doses overwhelm cellular antioxidant capacity, triggering oxidative damage and programmed cell death. Cancer cells maintain constitutively elevated basal RONS and frequently exhibit deficient antioxidant enzyme systems (SOD, CAT, PRX), positioning them closer to the oxidative stress threshold. CAP exploits this ‘redox vulnerability window’: doses mildly stressful to normal cells prove lethal to malignant cells. Selectivity has been demonstrated across various types of cancers and is extensible to other medical applications like hyper-proliferative dermatological conditions. **Abbreviations:** CAP, cold atmospheric plasma; RONS, reactive oxygen and nitrogen species; KEAP1, Kelch-like ECH-associated protein 1; Nrf2, nuclear factor erythroid 2-related factor 2; ARE, antioxidant response element; CAT, catalase; GSH, reduced glutathione; GCL, glutamate-cysteine ligase; GPx, glutathione peroxidase; GST, glutathione S-transferase; PRX, peroxiredoxin; H_2_O_2_, hydrogen peroxide; ONOO^−^, peroxynitrite; •OH, hydroxyl radical; •NO, nitric oxide.

The biological effects of CAP follow a non-monotonic, hormetic dose-response curve fundamentally determined by the concentration and composition of delivered RONS [[67], [68], [69]]. This section delineates two distinct dose windows: moderate-to-high doses that exploit oxidative stress for cytotoxic applications, and low-to-moderate doses that harness redox signaling for cytoprotective therapies [54] (Fig. 1).

### 2.1 Moderate-to-high for pro-oxidant effect

At pro-oxidant doses, CAP functions as a redox-modulating therapeutic that overwhelms endogenous antioxidant capacity, inducing oxidative damage and programmed cell death. This mechanistic paradigm forms the foundation for its application in proliferative disorders, particularly oncology. The underlying biochemical cascade is well-established: extracellular RONS, predominantly H_2_O_2_, peroxynitrite (ONOO^−^), and hydroxyl radicals (•OH), enter cells through multiple pathways, including aquaporin-mediated membrane penetration [70], cell membrane receptor interaction [71], and CAP-induced transient membrane permeabilization/permeation [[72], [73], [74], [75]], thereby elevating the intracellular oxidative burden [54,68]. This surge in oxidative stress triggers mitochondrial membrane depolarization, cytochrome *c* release, and subsequent activation of caspase-3/7, ultimately leading to apoptosis [43], a mechanistic cascade first established by Vandamme and colleagues in their pioneering study on plasma-induced cancer cell death [76]. Concurrently, DNA damage responses are initiated, marked by p53 phosphorylation, while lipid peroxidation compromises membrane integrity [77].

Recent advances have enabled more precise quantification of the thresholds governing these pro-oxidant effects. For instance, one study demonstrated that CAP-activated solution (CAS) exhibits mode- and dose-dependent variations in the production of RONS, the modification of amino acids (specifically tyrosine [Tyr] and tryptophan [Trp]), and the resulting antitumor efficacy and underlying mechanisms. Notably, treatment with the 4 kHz (oxygen) mode generated the Trp metabolites formylkynurenine (FKyn) and kynurenine (Kyn), which induced apoptosis in melanoma cells (Mel Im); in contrast, the 8 kHz (nitrogen) mode promoted the formation of nitrated derivatives of Trp and Tyr, leading to elevated p16 mRNA expression and increased senescence-associated β-galactosidase activity [78]. Furthermore, the intracellular abundance of RONS delivered to tumor tissue varies significantly across cell types. For instance, the critical H_2_O_2_ concentration required to achieve cancer cell eradication following exposure to 100 nM paclitaxel is 600 nM in MCF7 cells, compared to 1100 nM in HL-60 cells [79].

Selective cytotoxicity against malignant cells, arguably the most clinically significant manifestation of CAP's pro-oxidant action, operates through multiple synergistic mechanisms. Cancer cells are characterized by constitutively elevated basal ROS levels relative to their normal counterparts, positioning them closer to the oxidative stress threshold [57]. Furthermore, many tumors exhibit deficiencies in antioxidant enzyme systems, particularly catalase and glutathione peroxidase, thereby creating a therapeutic vulnerability [80]. CAP exploits this ‘redox vulnerability window’, doses that elicit only mild stress in normal cells become lethal to cancer cells [81].

Beyond direct cytotoxic effects, CAP-induced pro-oxidant stress has been shown to trigger immunogenic cell death (ICD) [82,83], a modality that effectively converts apoptotic cancer cells into an endogenous vaccine. Pioneering studies by Lin and colleagues [84] first demonstrated that CAP-treated cancer cells exposed calreticulin, released HMGB1 and ATP, and induced dendritic cell maturation, while subsequent work by Bekeschus and colleagues [85] further characterized the RONS-dependent nature of this response and established its immunogenicity in vivo. CAP-treated cancer cells release damage-associated molecular patterns (DAMPs), including surface-exposed calreticulin, secreted HMGB1, and extracellular ATP. These signals facilitate dendritic cell (DC) maturation and prime cytotoxic T lymphocyte responses [86,87]. This immunomodulatory dimension elevates CAP from a purely ablative intervention to a potential *in situ* vaccine adjuvant, offering a compelling strategy for integrating physical plasma into next-generation cancer immunotherapy regimens.

### 2.2 Low-to-moderate for anti-oxidant effect

At low-to-moderate doses, CAP exhibits a paradoxical hormetic effect: rather than inducing oxidative damage, it activates cellular antioxidant defense systems, thereby conferring protection against subsequent oxidative injury. This adaptive response, known as ‘preconditioning’ or ‘adaptive response’, represents an emerging therapeutic paradigm for degenerative diseases such as Alzheimer's disease, as well as inflammation-driven conditions where oxidative stress constitutes a core pathogenic mechanism.

The seminal demonstration of CAP-mediated neuroprotection by Tian and colleagues established quantitative parameters for this phenomenon [55]. Treatment of rat cortical neurons with a helium CAP jet for 4-10 s induced a 220% elevation of reduced glutathione (GSH), the principal intracellular thiol antioxidant, within 3 h post-treatment. This elevation suggests that micromolar-level peroxynitrite and 30-70 μM hydrogen peroxide generated by the CAP jet trigger known, dose-dependent pathways for activating cellular antioxidant defense [55]. Critically, this dosage range of reactive species aligns with CAP-generated 50 μM H_2_O_2_ previously shown to activate Nrf2 nuclear translocation and antioxidant response element (ARE) transcription in both immortalized human epidermal cells (HaCaT) and primary normal human epidermal keratinocytes (NHEK) [88].

One molecular circuitry underlying CAP-mediated antioxidant activation centers on the KEAP1-Nrf2 signaling pathway. Under basal conditions, KEAP1 (Kelch-like ECH-associated protein 1) facilitates Nrf2 (nuclear factor erythroid 2-related factor 2) ubiquitination and subsequent proteasomal degradation in the cytoplasm. Upon exposure to electrophilic RONS, RONS with electron-deficient centers that can covalently modify nucleophilic amino acid residues, specific cysteine residues in KEAP1 (Cys151, Cys273, Cys288) undergo modification, inducing conformational changes that impair Nrf2 binding [89,90]. Stabilized Nrf2 then translocates to the nucleus, where it heterodimerizes with small Maf proteins and binds to ARE sequences in the promoter regions of over 250 cytoprotective genes [91,92]. These include glutamate-cysteine ligase (both catalytic and modifier subunits), glutathione peroxidase, glutathione S-transferase, NAD(P)H:quinone oxidoreductase 1, and heme oxygenase-1 [[93], [94], [95]]. This coordinated transcriptional program underpins the observed elevation in cellular antioxidant capacity. This adaptive response exhibits characteristic temporal dynamics. Nrf2 target gene expression typically peaks between 6 and 12 h post-induction [96,97], with corresponding enzyme activities and GSH levels remaining elevated for 24-72 h [97]. This defined therapeutic window enables strategic prophylactic application of CAP prior to anticipated toxic insult, positioning it as a potential intervention for conditions involving predictable oxidative stress.

Beyond this preconditioning effect, which involves cellular adaptive responses wherein low-dose CAP activates Nrf2 signaling and upregulates endogenous antioxidant defenses over hours to days, the antioxidant applications of CAP extend to a mechanistically distinct modality: direct RONS scavenging. In this context, CAP-treated solutions and hydrogels retain stable RONS concentrations (primarily nitrite, nitrate, and residual hydrogen peroxide) that can neutralize pathological oxidative bursts through direct chemical reactions, independent of cellular signaling pathways. For instance, CAP-activated saline has been shown to reduce inflammation and ferroptosis in gingival fibroblasts within periodontitis models, demonstrating therapeutic utility in localized inflammatory conditions [98].

For instance, CAP-activated saline has been shown to reduce inflammation and ferroptosis in gingival fibroblasts within periodontitis models, demonstrating therapeutic utility in localized inflammatory conditions [68].

The biphasic dose-response relationship, pro-oxidant at high doses versus antioxidant at low doses, constitutes the central organizing principle for CAP dosing strategy. This hormetic framework underscores the necessity of precise dosimetry: the therapeutic window for antioxidant applications resides below the threshold for oxidative damage, yet above the threshold for meaningful biological effect. Determining the precise transition threshold for each device configuration, gas composition, and biological target remains the paramount operational challenge facing clinical translation of CAP-mediated antioxidant therapy. Addressing this challenge will require systematic investigation of dose-response relationships across diverse cell types and pathological contexts, ultimately enabling rational, mechanism-based treatment protocols that harness CAP's full therapeutic potential.

## 3 Critical parameters influencing CAP impact

**Fig. 2:**
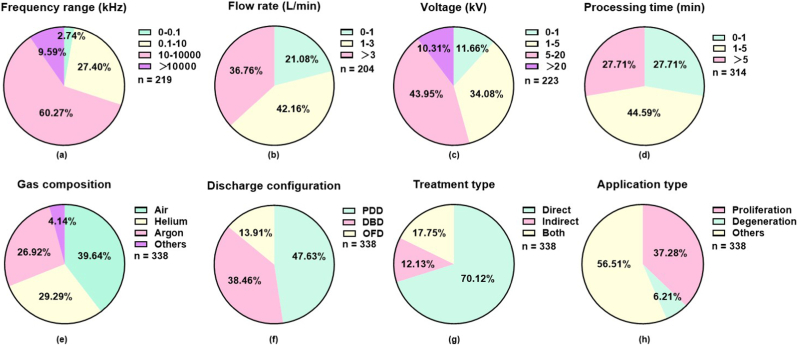
**Quantitative summary of recent (2020**–**2025) CAP studies categorized by key operational parameters.** Frequency distribution of studies according to **(a)** frequency range, **(b)** flow rate, **(c)** voltage, **(d)** processing time, **(e)** gas composition, **(f)** discharge configuration, **(g)** treatment type, and **(h)** biomedical application.

The dose delivered by CAP is not a single scalar quantity but a multidimensional vector determined by interacting physical and chemical parameters. This section systematically examines five critical control parameters, frequency, flow rate, voltage, processing time, and gas composition, elucidating their mechanistic influence on RONS generation and therapeutic efficacy (Fig. 2, Table 1).

**Table 1:** Key operational parameters influencing CAP dosage: a summary of studies from 2020 to 2025.

Discharge Type	Gas Type	Frequency (kHz)	Flow rate (L/min)	Voltage (kV)	Processing Time	Treatment Type	Treatment Formula	Study Type	Study Model	Disease Type	Disease Model	Ref
OFD	Argon	N/A	2	N/A	1 min	Direct	CAP	Phenotype	in ovo (chorioallantoic membrane)	Proliferation	Pancreatic cancer	[49]
PDD	Helium	52	1.21	1.4	0.17-3 min	Both	CAP	Phenotype; Mechanism	in vitro (cell)/in vivo (mice)	Proliferation	Neurofibromatosis	[99]
PDD	Helium	20	0.8	16	0-15 min	Both	CAP combination	Phenotype	in vitro (cell)/in vivo (mice)	Proliferation	Peritoneal cancer	[100]
DBD	Air	0.06	N/A	2.5	5-10 min	Direct	CAP	Phenotype; Mechanism	in vitro (cell)/in vivo (mice)	Proliferation	Psoriasis	[101]
DBD	Air	N/A	1	N/A	0.25-1 min	Direct	CAP	Phenotype; Mechanism	in vitro (cell)	Proliferation	Lung cancer	[102]
DBD	Helium	24	6	12	0.17-1 min (in vitro); 15 min (in vivo)	Direct	CAP	Phenotype; Mechanism	in vitro (cell)/in vivo (mice)	Proliferation	Solid cancer (lung, gastric, colon, renal)	[103]
DBD	Air	10	N/A	12	1-1.5 min	Direct	CAP	Phenotype; Mechanism	in vitro (cell)	Others	Dentistry	[104]
OFD	Air	N/A	N/A	14	N/A	Indirect	CAP combination	Phenotype; Mechanism	in vivo (mice)	Proliferation	Lymphatic sarcoma	[105]
PDD	Helium	N/A	5	7.5	0.25-1 min	Both	CAP	Phenotype	in vitro (cell)/in vivo (mice)	Others	Wound	[106]
PDD	Helium	10	3	5	10-30 s	Both	CAP	Phenotype	Others	Degeneration	Alzheimer's disease	[107]
OFD	Argon	1000	2	3	0.25-2 min	Both	CAP combination	Phenotype; Mechanism	in vitro (cell)	Proliferation	Prostate cancer	[108]
OFD	Helium	N/A	8.5	0.006	30 s	Both	CAP combination	Phenotype	in vitro (cell)/in vivo (mice)	Proliferation	Colorectal cancer	[109]
PDD	Argon	N/A	2	N/A	1.5 min	Both	CAP combination	Phenotype	in vitro (cell)/in vivo (mice)	Proliferation	Breast cancer	[110]
PDD	Argon	N/A	N/A	N/A	N/A	Direct	CAP	Phenotype; Mechanism	in vitro (cell)/in vivo (mice)	Proliferation	Thrombolysis	[111]
DBD	Air	8	2	3.5	2-5 min	Both	CAP	Phenotype; Mechanism	in vitro (cell)	Others	Wound	[112]
PDD	Air	83	2	1.6	5-20 min	Indirect	CAP	Phenotype; Mechanism	in vitro (cell)	Proliferation	Head & neck cancer	[113]
PDD	Helium	45	0.5	30	30 s	Direct	CAP	Phenotype; Mechanism	in vitro (skin)	Others	Wound	[114]
DBD	Air	N/A	N/A	N/A	1 min	Direct	CAP combination	Phenotype	in vitro (cell)	Others	Wound	[115]
DBD	Helium	31	1	10	1-7 min	Direct	CAP	Phenotype	in vivo (mice)	Others	Dentistry	[116]
PDD	Argon	2450000	8	N/A	2-20 s	Direct	CAP	Phenotype; Mechanism	in vitro (bacteria)	Others	Dentistry	[117]
PDD	Argon	50	2	4	3-6 min	Both	CAP combination	Phenotype; Mechanism	in vitro (bacteria)	Others	Antimicrobial therapy	[118]
DBD	Air	N/A	N/A	18	1-2 s	Direct	CAP	Phenotype; Mechanism	in vitro (cell)	Others	Dentistry	[119]
DBD	Air	N/A	N/A	N/A	5-40 s	Direct	CAP	Phenotype; Mechanism	in vitro (skin)	Others	Candidiasis	[120]
DBD	Helium/Oxygen	10	N/A	8.25	30 s (in vitro); 3 min (in vivo)	Direct	CAP	Phenotype; Mechanism	in vitro (cell)/in vivo (mice)	Degeneration	Atopic dermatitis	[121]
DBD	Air	1.22	N/A	N/A	0.5-10 min	Direct	CAP	Phenotype; Mechanism	in vitro (skin)	Others	Burn wound infection (*Pseudomonas aeruginosa*)	[122]
OFD	Argon	1700	5	6	5-30 s (in vitro); 10 min (in vivo)	Both	CAP	Phenotype; Mechanism	in vitro (cell)/in vivo (mice)	Others	SARS-CoV-2	[123]
DBD	Air	N/A	N/A	18	1 min	Direct	CAP	Phenotype; Mechanism	in vitro (cell)	Degeneration	Bone regeneration	[124]
OFD	Argon	23.5	1	7	2-5 min	Indirect	CAP combination	Phenotype; Mechanism	in vitro (bacteria)	Others	Wound	[125]
DBD	Air	N/A	N/A	N/A	2 min	Direct	CAP	Phenotype; Mechanism	in vitro (cell)/in vivo (mice)	Proliferation	Melanoma	[126]
DBD	Helium	32	2	13	2 min	Direct	CAP	Phenotype; Mechanism	in vitro (cell)	Others	*Candida albicans*	[127]
OFD	Helium	N/A	8.5	0.006	30-45 s	Both	CAP combination	Phenotype; Mechanism	in vitro (cell)/in vivo (mice)	Proliferation	Breast cancer	[128]
PDD	Helium	N/A	1.2	1.2	2-5 min	Indirect	CAP	Phenotype; Mechanism	in vitro (cell)	Proliferation	Prostate Cancer	[129]
OFD	Helium/Oxygen	2450000	2	N/A	N/A	Direct	CAP	Phenotype; Mechanism	in vitro (cell)	Others	Dentistry	[130]
DBD	Argon	40	10	4	0.5-3 min	Direct	CAP combination	Phenotype; Mechanism	in vitro (cell)	Proliferation	Oral squamous cell carcinoma	[131]
DBD	Helium	N/A	N/A	32	30 s	Direct	CAP	Phenotype; Mechanism	in vitro (cell)	Degeneration	Macrophages kill *Staphylococcus aureus*	[132]
OFD	N/A	1	N/A	4	1-2 min	Direct	CAP	Phenotype; Mechanism	in vitro (cell)	Proliferation	Breast cancer	[133]
PDD	Helium	10	1	1.24	4 min	Both	CAP	Phenotype; Mechanism	in vitro (cell)/in vivo (mice)	Proliferation	Breast cancer	[39]
OFD	Argon	N/A	3	N/A	5-60 s	Direct	CAP	Phenotype; Mechanism	in vitro (cell)	Proliferation	Osteosarcoma	[134]
PDD	Helium	N/A	N/A	N/A	30s	Direct	CAP	Phenotype; Mechanism	in vitro (skin)	Others	Wound	[135]
PDD	Air	N/A	N/A	15	0.5-30 min	Direct	CAP	Phenotype	in vitro (skin)	Others	Wound	[136]
DBD	Helium	10	N/A	32	30s	Direct	CAP	Phenotype; Mechanism	in vitro (cell)/in vivo (mice)	Others	Wound	[137]
PDD	Argon	2450000	4	N/A	2min	Direct	CAP	Phenotype; Mechanism	in vitro (cell)/in vivo (mice)	Proliferation	Dermatology (scleroderma; fibrosis)	[138]
PDD	Helium	10	1	1.24	4 min	Both	CAP	Phenotype; Mechanism	in vitro (cell)/in vivo (mice)	Degeneration	Viral vaccine manufacturing(IBRV、CPV、CDV)	[139]
DBD	Air	4	N/A	3.5	2 min	Direct	CAP combination	Phenotype	in vitro (cell)	Proliferation	Skin cancer	[47]
OFD	Helium/Oxygen	2450000	2	N/A	N/A	Direct	CAP	Phenotype; Mechanism	in vitro (cell)	Others	Dentistry	[140]
OFD	N/A	N/A	N/A	N/A	1-5 min	Direct	CAP	Phenotype; Mechanism	Others	Others	Dentistry	[141]
PDD	N/A	8	N/A	8	0-1 min	Both	CAP	Phenotype; Mechanism	in vitro (cell)/in vivo (mice)	Proliferation	Endometrial cancer	[142]
PDD	Air	N/A	7	9.64	0.13-2 min	Direct	CAP	Phenotype	in vitro (virus)	Others	SARS-CoV-2	[143]
PDD	Argon	N/A	2	N/A	0.5-2.5 min (in vitro); 2-15 min (in vivo)	Direct	CAP combination	Phenotype; Mechanism	in vitro (cell)/in vivo (mice)	Proliferation	Solid cancer (melanoma, colon)	[144]
DBD	Air	N/A	N/A	18	1 min	Direct	CAP	Phenotype; Mechanism	in vitro (cell)	Others	Wound	[145]
PDD	Helium	52	3	10	0.17-2 min	Direct	CAP	Phenotype	in vitro (cell)	Others	Wound	[146]
PDD	Air	N/A	N/A	N/A	0.25-2 min	Direct	CAP	Phenotype; Mechanism	in vitro (cell)/in vivo (mice)	Proliferation	Solid cancer (colorectal, lung)	[147]
OFD	Argon	1700	5	6	5-30 s (in vitro); 10 min (in vivo)	Both	CAP combination	Phenotype; Mechanism	in vitro (cell)/in vivo (mice)	Proliferation	Breast cancer	[148]
OFD	Argon	N/A	5	N/A	1 min	Direct	CAP	Phenotype	Others	Others	Dentistry	[149]
PDD	Helium	12	0.12	N/A	30 s	Direct	CAP	Phenotype; Mechanism	in vitro (cell)/in vivo (mice)	Others	Wound	[150]
PDD	Helium/Air	N/A	2.35	6.6	5 min	Direct	CAP	Phenotype; Mechanism	in vivo (rabbit)	Others	MRSA	[151]
DBD	Air	1.22	N/A	N/A	10 s	Direct	CAP	Phenotype; Mechanism	in vivo (mouse)	Degeneration	Diabetic wounds	[152]
PDD	Air	8	6.8	0.06	10 s (in vitro); 4 min (in vivo)	Direct	CAP	Phenotype; Mechanism	in vitro (cell)/in vivo (mice)	Proliferation	Solid cancer (breast, melanoma)	[153]
PDD	Argon	20	3	2.77	0.08-1 min	Direct	CAP	Phenotype; Mechanism	in vitro (cell)/in vivo (mice)	Proliferation	Oral squamous cell carcinoma	[154]
PDD	Argon/Oxygen	21.7	1.9	N/A	1-2 min	Both	CAP	Phenotype; Mechanism	in vitro (cell)/in vivo (mice)	Proliferation	Myeloid leukemia	[155]
PDD	Helium	10	1	1.24	4 min	Both	CAP	Phenotype; Mechanism	in vitro (cell)/in vivo (mice)	Others	Viral vaccine manufacturing	[156]
PDD	Air	N/A	5	20	30 min	Indirect	CAP	Phenotype; Mechanism	in vitro (virus)/in vitro (cell)	Others	SARS-CoV-2, Influenza	[157]
OFD	Argon/Helium	N/A	N/A	N/A	3 min	Direct	CAP	Phenotype; Mechanism	Others	Others	Dentistry	[158]
PDD	Air	13.27	N/A	3.8	1-30 min	Direct	CAP	Phenotype; Mechanism	Others	Others	Environment	[159]
PDD	Argon	23.5	6	8	1-5 min	Direct	CAP	Phenotype	in vitro (biofilm)	Others	*Staphylococcus aureus*, *Pseudomonas aeruginosa*, *Candida albicans*	[160]
PDD	Air	0.05	N/A	0.23	N/A	Indirect	CAP combination	Phenotype; Mechanism	in vivo (mice)	Degeneration	Aging associated neurodegeneration	[161]
DBD	Air	N/A	N/A	3.92	10-40 s	Direct	CAP	Phenotype; Mechanism	in vitro (bacteria)	Others	*Shigella flexneri*	[162]
DBD	Air	7	N/A	N/A	1-5 min	Both	CAP combination	Phenotype; Mechanism	in vitro (cell)	Proliferation	Esophageal cancer	[163]
DBD	Air	8	N/A	3.5	2-5 min	Both	CAP	Phenotype	in vitro (cell)	Proliferation	Melanoma	[78]
DBD	Air	0.4	N/A	27	0-10 min	Direct	CAP	Phenotype	in vitro (biofilm)	Others	Fungal keratitis	[164]
DBD	Helium	50	9	3.5	1 min	Direct	CAP combination	Phenotype; Mechanism	in vitro (cell)	Proliferation	Lung cancer	[165]
PDD	Helium	10	4	5	0.25-2 min	Direct	CAP combination	Phenotype; Mechanism	in vitro (cell)	Proliferation	Glioblastoma	[166]
DBD	Helium	20	3	4	2-4 min	Indirect	CAP combination	Phenotype; Mechanism	in vitro (fungal)	Others	Biotechnology	[167]
DBD	Air	N/A	N/A	N/A	10 min	Direct	CAP	Phenotype; Mechanism	in vitro (cell)	Others	*Aspergillus brasiliensis*	[168]
PDD	Air	50	N/A	N/A	0.25-2 min	Direct	CAP combination	Phenotype; Mechanism	in vitro (cell)/in vivo (mice)	Proliferation	Head and neck cancer	[37]
DBD	Air	N/A	N/A	18	30 s (in vitro); 1 min(in vivo)	Direct	CAP	Phenotype	in vitro (cell)	Others	Wound	[169]
PDD	Air	N/A	3	2.6	5-10 min	Direct	CAP	Phenotype	in vitro (bacteria)	Others	*Staphylococcus aureus*	[170]
OFD	Argon	1000	5	6	1 min	Direct	CAP	Phenotype; Mechanism	in vitro (biofilm)	Others	Dentistry	[171]
OFD	Argon/Helium	1000	3	3	9 min	Both	CAP combination	Phenotype; Mechanism	in vitro (cell)	Proliferation	Glioblastoma	[172]
OFD	Argon	N/A	1	N/A	5 min	Indirect	CAP combination	Phenotype; Mechanism	in vitro (cell)/in vivo (mice)	Proliferation	Glioblastoma	[82]
PDD	Air	50	N/A	N/A	0.25-2 min	Direct	CAP	Phenotype; Mechanism	in vitro (cell)/in vivo (mice)	Proliferation	Hepatocellular cancer	[173]
DBD	Air	28	N/A	10	0.25-1 min	Indirect	CAP combination	Phenotype	in vitro (cell)	Proliferation	Breast cancer	[174]
OFD	Argon	1000	4.1	3	30s	Direct	CAP	Phenotype	in vitro (bacteria)	Others	Canine Bacterial Keratitis	[175]
OFD	N/A	1	N/A	4	0.25-2 min	Direct	CAP	Phenotype; Mechanism	in vitro (cell)	Proliferation	Bladder cancer	[176]
OFD	Argon/Helium	39.6	5	9.78	3 min(in vitro); 5 min(in vivo)	Direct	CAP	Phenotype; Mechanism	in vitro (cell)	Proliferation	Colon cancer	[177]
DBD	Air	62	2.7	16.4	1-5 min	Direct	CAP	Phenotype; Mechanism	in vivo (porcine)	Others	Axillary odor caused by infection	[178]
DBD	Air	7.5	1.5	N/A	0.25-2 min	Direct	CAP	Phenotype; Mechanism	in vitro (cell)/in vivo (mice)	Others	Safety	[179]
DBD	Air	N/A	N/A	75	50 s	Direct	CAP combination	Phenotype; Mechanism	in vitro (cell)	Proliferation	Glioblastoma	[180]
PDD	Argon	N/A	N/A	N/A	40 s	Direct	CAP combination	Phenotype; Mechanism	in vitro (cell)	Proliferation	Melanoma	[181]
OFD	Argon	1000	4.1	3	2 min(in vitro); 5 min(in vivo)	Direct	CAP	Phenotype	in vivo (canine)	Others	Canine bacterial keratitis	[182]
DBD	Air	N/A	N/A	31.2	2-6 min	Both	CAP	Phenotype; Mechanism	in vitro (cell)	Proliferation	Breast cancer	[183]
OFD	Argon	1000	3	3	0.25-2 min	Both	CAP combination	Phenotype; Mechanism	in vitro (cell)/in vivo (mice)	Proliferation	Osteosarcoma	[184]
DBD	Air	7	1	N/A	1-5 min	Direct	CAP	Phenotype; Mechanism	in vitro (cell)	Proliferation	Esophageal squamous cancer	[185]
PDD	Argon	48	2	9	0.5-2 min	Direct	CAP	Phenotype; Mechanism	in vitro (bacteria)	Others	Wound	[186]
PDD	Helium	13560	1.4	N/A	0-10 min	Both	CAP	Phenotype; Mechanism	in vitro (cell)	Others	Wound	[187]
DBD	Helium	20	3	10	1-5 min	Direct	CAP	Phenotype; Mechanism	in vitro (cell)/in vivo (mice)	Proliferation	Neuroblastoma	[188]
PDD	Air	N/A	N/A	4	0.5-3 h	Indirect	CAP	Phenotype	in vitro (bacteria)	Others	Multidrug-resistant bacteria(CRAB, CRPA, MRSA, CRKP)	[189]
DBD	Air	4	N/A	3.5	5 min	Indirect	CAP	Phenotype; Mechanism	in vitro (cell)	Degeneration	Neutrophil function and oxidative burst	[190]
PDD	Air	50	N/A	N/A	0.17-2 min	Direct	CAP	Phenotype; Mechanism	in vitro (bacteria)	Others	Periodontitis, Peri-implantitis	[191]
PDD	Helium	31.7	2	12	1-7 min	Direct	CAP	Phenotype	in vitro (biofilm)	Others	Dentistry	[192]
PDD	Air	83	2	1.6	5-20 min	Indirect	CAP	Phenotype; Mechanism	in vitro (cell)	Proliferation	Head and neck cancer	[193]
PDD	Argon	351	1	N/A	1 min-50 min	Direct	CAP	Phenotype; Mechanism	Others	Others	Environment	[194]
PDD	Helium	15	10	5	2-8 min	Indirect	CAP	Phenotype	in vitro (fungal)	Others	Biotechnology	[195]
PDD	Helium	13	5	0.22	0.5-1.5 min	Direct	CAP combination	Phenotype; Mechanism	in vitro (cell)/in vivo (mice)	Proliferation	Melanoma	[196]
DBD	Air	N/A	1	N/A	2 min	Both	CAP	Phenotype; Mechanism	in vitro (cell)	Proliferation	Melanoma	[197]
PDD	Helium	52	1.21	1.4	0-3 min	Direct	CAP	Phenotype; Mechanism	in vitro (cell)	Proliferation	Chordoma	[198]
DBD	Argon	13560	1	N/A	0.5-2 min	Direct	CAP	Phenotype; Mechanism	in vitro (cell)	Degeneration	Cell adhesion (Bone marrow mesenchymal stem cells)	[199]
DBD	Air	N/A	N/A	N/A	4.5 min	Direct	CAP	Phenotype	in vitro (skin)	Others	Wound	[200]
OFD	Argon/Oxygen	N/A	3	N/A	30 s	Direct	CAP	Phenotype; Mechanism	Others	Others	Dentistry	[201]
PDD	Helium	N/A	2	13	2.5-5 min	Direct	CAP	Phenotype; Mechanism	in vitro (biofilm)	Others	*Candida albicans*	[202]
DBD	Air	N/A	N/A	75	10-40 s	Both	CAP combination	Phenotype; Mechanism	in vitro (cell)	Proliferation	Glioblastoma	[203]
PDD	Helium	9.5	3.17	5.33	N/A	Both	CAP	Phenotype	in vitro (cell)	Proliferation	Glioblastoma	[63]
PDD	Helium	13	5	0.22	0.5-1.5 min	Direct	CAP combination	Phenotype; Mechanism	in vitro (cell)	Proliferation	Melanoma	[204]
DBD	Argon	N/A	1.5	3.7	N/A	Direct	CAP	Phenotype; Mechanism	in vitro (bacteria)	Others	*Pseudomonas fluorescens*	[205]
PDD	Argon	N/A	2.5	25	40 s	Direct	CAP	Phenotype; Mechanism	in vitro (cell)	Proliferation	Melanoma	[206]
DBD	Helium	83	3	2.3	N/A	Direct	CAP	Phenotype	in vitro (bacteria)	Others	*Pseudomonas aeruginosa*	[207]
PDD	Helium	10	1	1.24	3-4 min	Both	CAP combination	Phenotype; Mechanism	in vitro (cell)/in vivo (mice)	Proliferation	Colorectal cancer	[208]
PDD	Helium	N/A	8.5	0.006	0.5-8 min	Both	CAP	Phenotype; Mechanism	in vitro (cell)	Proliferation	Melanoma	[32]
PDD	Argon	N/A	3	15	10-50 s	Direct	CAP combination	Phenotype; Mechanism	in vitro (cell)	Proliferation	Cervical cancer	[209]
DBD	Air	20	N/A	8	0.5-5 min	Direct	CAP	Phenotype; Mechanism	in vitro (virus)	Others	SARS-CoV-2	[210]
DBD	Argon	12.89	1.5	0.38	10 min	Direct	CAP	Phenotype; Mechanism	in vitro (cell)	Proliferation	Breast cancer	[211]
PDD	Helium/Oxygen	14	N/A	8	10 min	Direct	CAP	Phenotype; Mechanism	Others	Others	Dentistry	[212]
DBD	Air	N/A	N/A	N/A	1.5 min	Direct	CAP	Phenotype	in vitro (skin)	Degeneration	Rosacea	[213]
DBD	Helium	2	2	7	0.25-5 min	Both	CAP combination	Phenotype; Mechanism	in vitro (cell)/in vivo (mice)	Proliferation	Melanoma	[214]
PDD	Argon/Oxygen/Nitrogen	9	10	9	5 min	Direct	CAP	Phenotype; Mechanism	in vitro (cell)/in vivo (mice)	Proliferation	Breast Cancer	[215]
PDD	Nitrogen	N/A	1	N/A	1.17 min	Direct	CAP	Phenotype; Mechanism	in vitro (cell)	Others	Dentistry	[216]
PDD	Air	N/A	N/A	N/A	1.5 min	Direct	CAP	Phenotype	in vivo (mice)	Others	Wound	[217]
PDD	Argon/Helium	12	0.12	N/A	30 s	Direct	CAP	Phenotype; Mechanism	in vitro (cell)/in vivo (porcine)	Others	Wound	[218]
PDD	Helium	10	2	10	20 s	Direct	CAP	Phenotype; Mechanism	in vitro (cell)/in vivo (mice)	Proliferation	Multiple myeloma	[219]
PDD	Helium	N/A	0.5	N/A	1-7 min	Direct	CAP	Phenotype	in vitro (bacteria)	Others	Dentistry	[220]
PDD	Argon	950	2.3	3	1 min	Direct	CAP	Phenotype	Others	Others	Dentistry	[221]
PDD	Argon	N/A	N/A	1.2	0.5-5 min	Direct	CAP	Phenotype	Others	Others	Dentistry	[222]
DBD	Air	N/A	1	N/A	1 min	Direct	CAP	Phenotype	in ovo (chorioallantoic membrane)	Proliferation	Solid cancer(liver cancer)	[223]
DBD	Helium	52	9	3.5	1 min	Direct	CAP	Phenotype; Mechanism	in vitro (cell)	Proliferation	Lung cancer	[224]
OFD	Argon	N/A	3	N/A	5-10 min	Direct	CAP	Phenotype; Mechanism	Others	Others	Dentistry	[225]
PDD	Argon	10	2	10	1.33 min	Direct	CAP	Phenotype; Mechanism	in vitro (cell)	Proliferation	Breast cancer	[226]
PDD	Argon	18	2	11	1.5 min	Direct	CAP	Phenotype; Mechanism	in vitro (cell)	Proliferation	Myeloid leukemia	[227]
PDD	Air	N/A	0.6	N/A	N/A	Both	CAP	Phenotype; Mechanism	in vitro (cell)	Proliferation	Melanoma	[228]
PDD	Helium	50	3	N/A	1 min	Direct	CAP	Phenotype	Others	Others	Dentistry	[229]
PDD	Helium	N/A	N/A	N/A	40 s	Direct	CAP combination	Phenotype; Mechanism	in vitro (cell)	Proliferation	Melanoma	[230]
PDD	Helium	N/A	3	N/A	3-6 min	Direct	CAP	Phenotype; Mechanism	in vitro (cell)	Proliferation	Breast cancer	[231]
PDD	Argon/Helium	20	2	4.5	1.17-3.37 min	Both	CAP	Phenotype; Mechanism	in vitro (cell)/in vivo (mice)	Proliferation	Glioblastoma	[232]
PDD	Air	25	N/A	24	15-45 min	Indirect	CAP	Phenotype; Mechanism	in vivo (mice)	Others	Acute Peritonitis	[233]
PDD	Helium	30	4	4	1-10 min	Direct	CAP	Phenotype; Mechanism	Others	Others	Biotechnology	[234]
PDD	Helium	N/A	N/A	N/A	3 min	Direct	CAP	Phenotype	Others	Others	Peri-implantitis	[235]
PDD	Air	42	1	2.2	3.33 min	Indirect	CAP	Phenotype; Mechanism	in vitro (cell)	Others	Wound	[236]
OFD	Argon	N/A	3	N/A	0.083-1 min	Direct	CAP	Phenotype; Mechanism	in vitro (cell)	Proliferation	Actinic keratosis	[237]
OFD	Argon	N/A	3	N/A	0.25-2 min	Both	CAP combination	Phenotype; Mechanism	in vitro (cell)	Proliferation	Bone cancer	[238]
OFD	Argon	N/A	3	N/A	0.5-4 min	Indirect	CAP	Phenotype; Mechanism	in vitro (cell)	Proliferation	Osteosarcoma	[238]
PDD	Helium	8.8	1	1.4	1-5 min	Both	CAP	Phenotype; Mechanism	in vitro (cell)/in vivo (mice)	Proliferation	Breast cancer	[70]
PDD	Helium	65	N/A	N/A	1 min	Direct	CAP combination	Phenotype	Others	Others	Dentistry	[239]
PDD	Helium	20	2	4	N/A	Direct	CAP combination	Phenotype	Others	Others	Dentistry	[240]
PDD	Helium	18	1.6	12	0.5-1 min	Both	CAP	Phenotype; Mechanism	in vitro (cell)	Proliferation	Colorectal cancer	[241]
PDD	Helium	10	1	11	0.17-2 min	Both	CAP	Phenotype; Mechanism	in vitro (cell)	Others	Wound	[242]
OFD	Argon	11000	6	6	10 s	Direct	CAP	Phenotype; Mechanism	in vitro (cell)	Proliferation	Hepatocellular cancer	[243]
DBD	Air	8	N/A	3.5	2 min	Direct	CAP combination	Phenotype; Mechanism	in vitro (cell)/in vivo (mice)	Proliferation	Squamous cell cancer	[244]
DBD	Helium	50	2	1.7	0.17-1.5 min	Direct	CAP	Phenotype; Mechanism	in vitro (cell)	Proliferation	Melanoma	[81]
DBD	Helium	100	2	2	0.5-4 min	Direct	CAP	Phenotype; Mechanism	in vitro (cell)	Others	Gingivitis	[245]
DBD	Air	N/A	N/A	6.495	1-3 min	Direct	CAP	Phenotype; Mechanism	in vitro (cell)	Proliferation	Colorectal cancer	[246]
DBD	Air	N/A	1	N/A	5 min	Direct	CAP combination	Phenotype; Mechanism	in vitro (cell)	Proliferation	Breast cancer	[247]
PDD	Air	1.4	N/A	5.74	1-3 min	Direct	CAP	Phenotype	in vitro (cell)	Others	Wound	[248]
PDD	Helium	1.4	N/A	10	30 s	Direct	CAP combination	Phenotype	Others	Others	Dentistry	[249]
DBD	Argon	N/A	2	3	0.25-4 min	Both	CAP combination	Phenotype; Mechanism	in vitro (cell)	Proliferation	Breast cancer	[250]
DBD	Air	5	N/A	3.2	1-5 min	Direct	CAP	Phenotype; Mechanism	in vitro (cell)/in vivo (mice)	Proliferation	Colorectal cancer	[251]
PDD	Helium	N/A	3	N/A	1-7 min	Direct	CAP	Phenotype; Mechanism	in vitro (cell)	Proliferation	Soft tissue sarcoma (fibrosarcoma, synovial Sarcoma, rhabdomyosarcoma, liposarcoma)	[252]
DBD	Air	N/A	0.5	N/A	5 min	Direct	CAP	Phenotype; Mechanism	in vitro (cell)	Others	Pneumonia	[253]
PDD	Helium/Oxygen	15000	1	0.01	1.5-3.5 min	Direct	CAP	Phenotype; Mechanism	in vitro (fungal)	Others	*Trichophyton rubrum*	[254]
PDD	Air	N/A	1	N/A	4 min	Direct	CAP combination	Phenotype; Mechanism	Others	Others	*Listeria monocytogenes*, *Escherichia coli*	[255]
PDD	Argon	100	5	0.000001	20 min	Direct	CAP combination	Phenotype	Others	Others	Dentistry	[256]
DBD	Air	0.3	N/A	0.04	1.5 min	Direct	CAP	Phenotype	in vitro (skin)	Others	Wound	[257]
PDD	Nitrogen	13560	N/A	N/A	4-8 min	Direct	CAP	Phenotype; Mechanism	Others	Degeneration	Mouse embryo fibroblasts	[258]
PDD	Helium	N/A	3	N/A	N/A	Direct	CAP	Phenotype; Mechanism	in vivo (porcine, rat)	Others	Wound	[259]
PDD	Argon	18	0.7	18	5 min	Both	CAP combination	Phenotype; Mechanism	in vitro (cell)	Proliferation	Cervical cancer	[260]
PDD	Helium	10	1	1.24	4 min	Both	CAP	Phenotype; Mechanism	in vitro (cell)/in vivo (mice)	Proliferation	Breast cancer	[261]
DBD	Air	0.4	N/A	27	1-7 min	Direct	CAP combination	Phenotype; Mechanism	in vitro (fungal)	Others	*Aspergillus flavus*, *Fusarium keratoplasticum*	[262]
DBD	Helium	12.5	4	10	4-12 min	Direct	CAP	Phenotype; Mechanism	in vitro (cell)	Proliferation	Glioblastoma	[263]
PDD	Helium	N/A	3	N/A	1-7 min	Direct	CAP combination	Phenotype; Mechanism	in vitro (cell)	Proliferation	Cholangiocarcinoma	[264]
PDD	Helium	10	2	8	40 s	Direct	CAP	Phenotype; Mechanism	in vitro (cell)	Proliferation	Myeloid Leukemia	[265]
DBD	Argon	13560	4	N/A	0.5-3 min	Direct	CAP	Phenotype; Mechanism	in vitro (cell)	Proliferation	Breast cancer	[266]
PDD	Argon	N/A	2	4	0.083-3 min	Direct	CAP	Phenotype; Mechanism	in vitro (cell)	Proliferation	Melanoma	[267]
PDD	Helium	N/A	3	N/A	5 min	Direct	CAP combination	Phenotype; Mechanism	in vitro (cell)/in vivo (mice)	Proliferation	Breast cancer	[268]
PDD	Air	25	N/A	20	5-15 min	Indirect	CAP combination	Phenotype; Mechanism	in vitro (cell)	Proliferation	Glioblastoma	[269]
PDD	Air	86	2	1.7	0.5-1 min	Direct	CAP	Phenotype	in vitro (cell)	Proliferation	Head and neck cancer	[270]
OFD	Argon	N/A	3	N/A	30 s	Direct	CAP	Phenotype	in vitro (bacteria)	Others	*Streptococcus pyogenes*	[271]
PDD	Argon/Air	21	2	6	0.25-5 min	Direct	CAP	Phenotype; Mechanism	in vitro (bacteria)	Others	*Escherichia coli*	[272]
OFD	Air	1	N/A	10	0.25-5 min	Direct	CAP	Phenotype; Mechanism	in vitro (biofilm)	Others	*Pseudomonas aeruginosa*, *Staphylococcus aureus*	[273]
DBD	Air	20	1	0.15	20 min	Indirect	CAP	Phenotype; Mechanism	in vitro (cell)	Degeneration	Human uterosactral ligament fibroblast	[274]
DBD	Helium	80	2	N/A	10 min	Indirect	CAP	Phenotype; Mechanism	in vivo (mice)	Others	Wound	[275]
DBD	Air	1.22	N/A	N/A	5-10 s	Direct	CAP	Phenotype; Mechanism	in vivo (porcine)	Others	Wound	[276]
OFD	Argon	N/A	3.5	N/A	0.083-1 min	Both	CAP combination	Phenotype; Mechanism	in vitro (cell)	Proliferation	Chondrosarcoma	[277]
PDD	Argon/Helium	39.6	4	11	3 min	Both	CAP	Phenotype; Mechanism	in vitro (organoid)	Proliferation	Colon cancer	[278]
DBD	Helium	100	3.8	0.78	3-3.5 min	Direct	CAP	Phenotype	in vitro (fungal)	Others	*Candida albicans* infection (oral candidiasis)	[279]
PDD	Helium	12.5	4	0.01	0.5-1.5 min	Both	CAP combination	Phenotype; Mechanism	in vitro (cell)/in vivo (mice)	Proliferation	Glioblastoma	[280]
OFD	Argon	2450000	2	N/A	1.5 min	Direct	CAP combination	Phenotype; Mechanism	in vitro (cell)/in vivo (mice)	Proliferation	Oral squamous cell carcinoma	[281]
OFD	Argon	1000	5	N/A	1 min	Direct	CAP	Phenotype; Mechanism	in vitro (cell)/in vivo (skin)	Others	Wound	[282]
PDD	Helium	13560	1	N/A	0.017-3 min	Direct	CAP combination	Phenotype; Mechanism	in vitro (cell)	Proliferation	Oral squamous cell carcinoma	[283]
DBD	Air	0.06	N/A	15	1-30 min	Direct	CAP	Phenotype; Mechanism	in vitro (cell)	Others	*Candida albicans*, *Staphylococcus aureus*	[284]
DBD	Helium	15000	1	12	0-6 min	Direct	CAP	Phenotype; Mechanism	in vitro (biofilm)	Others	*Staphylococcus aureus*	[285]
DBD	Helium	N/A	4	0.02188	10-30 min	Direct	CAP	Phenotype; Mechanism	in vitro (biofilm)	Others	*Listeria monocytogenes*, Salmonella typhimurium	[286]
DBD	Air	4	N/A	3.5	2 min	Direct	CAP combination	Phenotype; Mechanism	in vitro (cell)/in vivo (mice)	Proliferation	Skin cancer	[287]
PDD	Helium	100	1.85	N/A	30 s	Direct	CAP	Phenotype	Others	Others	Dentistry	[288]
DBD	Air	4	0.5	3.5	10 min	Direct	CAP	Phenotype; Mechanism	in vivo (mice)	Others	Pneumonia	[289]
DBD	Air	N/A	N/A	0.016	0.5-1 min	Both	CAP	Phenotype; Mechanism	in vitro (bacteria)	Others	*Pseudomonas aeruginosa*	[290]
PDD	Argon	1700	0.5	6	3 min	Direct	CAP	Phenotype	in vivo (mice)	Degeneration	Atopic dermatitis	[291]
PDD	Helium	N/A	1	0.012	4 min	Both	CAP	Phenotype	in vitro (cell)	Proliferation	Breast cancer	[292]
PDD	Air	N/A	N/A	N/A	2-6 min	Both	CAP	Phenotype; Mechanism	in vitro (cell)	Others	Wound	[293]
OFD	Argon	N/A	4	N/A	0.5-1.5 min	Direct	CAP	Phenotype; Mechanism	in vivo (mice)	Others	Wound	[294]
OFD	Argon	N/A	4	N/A	0.17-1.5 min	Direct	CAP	Phenotype; Mechanism	in vitro (tissue)	Others	Wound	[295]
OFD	Argon	2450000	1.9	N/A	1-3 min	Indirect	CAP	Phenotype; Mechanism	in vitro (cell)/in vivo (mice)	Proliferation	Lung cancer	[296]
OFD	Argon	1100	3	0.065	10 s	Direct	CAP	Phenotype; Mechanism	in vitro (cell)	Proliferation	Fibroblast	[297]
PDD	Argon	N/A	1.6	N/A	N/A	Both	CAP	Phenotype; Mechanism	in vitro (cell)	Proliferation	Breast cancer	[298]
DBD	Argon/Helium	N/A	N/A	N/A	1 min	Direct	CAP	Phenotype; Mechanism	in vitro (cell)	Proliferation	Breast cancer	[299]
DBD	Air	10	N/A	N/A	20 s	Direct	CAP	Phenotype	Others	Others	Dentistry	[300]
PDD	Air	N/A	N/A	N/A	1-21 min	Indirect	CAP	Phenotype; Mechanism	in vitro (cell)	Proliferation	Solid cancer (oral cancer, osteosarcoma, glioblastoma)	[301]
PDD	Argon	100	5	10	2 min	Direct	CAP	Phenotype	in vitro (biofilm)	Others	Dentistry	[302]
DBD	Air	N/A	N/A	N/A	10 min	Direct	CAP	Phenotype; Mechanism	in vitro (cell)	Others	*Aspergillus brasiliensis*	[303]
DBD	Air	10	5	12	15 min	Indirect	CAP	Phenotype; Mechanism	in vitro (cell)/in vivo (mice)	Proliferation	Melanoma	[304]
DBD	Air	0.05	6.8	5	N/A	Indirect	CAP	Phenotype; Mechanism	in vivo (mice)	Degeneration	Body metabolism	[305]
PDD	N/A	50	3	N/A	1 min	Direct	CAP combination	Phenotype	in vitro (bacteria)	Others	*Streptococcus sanguinis*	[306]
PDD	Helium	N/A	5	N/A	0.5-1.5 min	Direct	CAP combination	Phenotype; Mechanism	in vitro (cell)	Proliferation	Glioblastoma	[307]
PDD	Helium/Oxygen	35	N/A	6	0.5-3 min	Direct	CAP combination	Phenotype; Mechanism	in vitro (cell)	Proliferation	Cervical cancer	[308]
PDD	Air	N/A	N/A	0.012	10-40 min	Direct	CAP	Phenotype; Mechanism	Others	Others	Environment	[309]
DBD	Air	6	0.5	13	0.5-3 min	Both	CAP	Phenotype; Mechanism	in vitro (cell)	Proliferation	Osteosarcoma	[310]
DBD	Helium	50	7	8	2 min	Direct	CAP	Phenotype	in vivo (bovine)	Others	Dentistry	[311]
PDD	Air	62.5	N/A	3.44	30 s	Direct	CAP	Phenotype	in vitro (skin)	Degeneration	Skin aging	[312]
PDD	Helium	N/A	2	N/A	5-10 min	Direct	CAP	Phenotype; Mechanism	in vitro (skin)	Degeneration	Irritant contact dermatitis	[313]
PDD	Argon/Helium	39.6	5	9.78	3-5 min	Direct	CAP	Phenotype; Mechanism	in vitro (cell)	Proliferation	Colon cancer	[278]
PDD	Argon	N/A	N/A	N/A	30 s	Indirect	CAP combination	Phenotype	in vitro (cell)	Others	Wound	[11]
DBD	Air	0.7	N/A	30	10 s	Direct	CAP	Phenotype; Mechanism	in vivo (mice)	Proliferation	Melanoma	[314]
DBD	Air	0.6	N/A	17	1-30 min	Direct	CAP	Phenotype; Mechanism	in vivo (cell)	Others	Osteitis, osteomyelitis	[315]
DBD	Air	1.22	N/A	N/A	0.17-10 min	Direct	CAP	Phenotype; Mechanism	in vivo (mouse)	Others	Wound	[9]
PDD	Air	38	N/A	1.45	3 min	Indirect	CAP	Phenotype	in vitro (skin)	Others	Wound	[10]
PDD	Argon	N/A	2	8	5 min	Direct	CAP	Phenotype; Mechanism	in vitro (virus)	Others	Coronavirus disinfection (SARS-CoV-2 variants, swine coronaviruses)	[316]
PDD	Argon	13560	N/A	N/A	10-30 s	Direct	CAP combination	Phenotype; Mechanism	in vitro (virus)	Others	Respiratory infection(*Pseudomonas aeruginosa*, rhinovirus)	[317]
PDD	Helium	N/A	0.5	N/A	1-7 min	Direct	CAP	Phenotype	in vitro (bacteria)	Others	Dentistry	[318]
PDD	Air	25	1	7	8-10 min	Direct	CAP	Phenotype; Mechanism	Others	Degeneration	Alzheimer's disease	[319]
OFD	Argon	1000	4.1	6	2-5 min	Direct	CAP	Phenotype; Mechanism	in vivo (human)	Others	Wound	[12]
PDD	Air	N/A	N/A	30	5-20 min	Indirect	CAP	Phenotype; Mechanism	in vitro (cell)	Proliferation	Hepatocellular cancer	[320]
DBD	Air	20	N/A	5.2	5 min	Direct	CAP combination	Phenotype; Mechanism	in vivo (mice)	Degeneration	Skin aging	[321]
PDD	Argon	N/A	5	7.5	0.25-1 min	Both	CAP	Phenotype	in vitro (cell)/in vivo (mice)	Proliferation	Melanoma	[322]
OFD	Argon	1000	3	3	0.083-1 min	Both	CAP	Phenotype; Mechanism	in vitro (cell)	Proliferation	Renal cell cancer	[323]
PDD	N/A	N/A	N/A	3	1-30 min	Both	CAP combination	Phenotype; Mechanism	Others	Others	Environment	[18]
DBD	Argon/Air	0.02	3	25.6	0.33-5 min	Direct	CAP	Phenotype; Mechanism	Others	Others	Environment	[19]
PDD	Helium	20	4	6	2-6 min	Indirect	CAP	Phenotype; Mechanism	in vitro (cell)	Proliferation	Melanoma	[34]
DBD	Air	4	N/A	3.5	2 min	Direct	CAP	Phenotype; Mechanism	in vivo (mice)	Degeneration	Radiation dermatitis	[324]
DBD	Helium	24	2	12	30 s	Both	CAP	Phenotype; Mechanism	in vitro (cell)	Proliferation	Lung cancer	[325]
DBD	N/A	18	35	20	15 min	Direct	CAP	Phenotype; Mechanism	in vitro (bacteria)	Others	*Escherichia coli*, Klebsiella	[326]
PDD	Argon	N/A	N/A	10	30 s	Direct	CAP	Phenotype	in vitro (cell)	Others	Wound	[13]
PDD	Air	42	1	2.2	0.5-2 min	Indirect	CAP	Phenotype; Mechanism	in vitro (cell)	Others	Wound	[14]
PDD	Air	13560	1	20	5-25 min	Indirect	CAP	Phenotype; Mechanism	in vitro (cell)/in vivo (mice)	Proliferation	Osteosarcoma	[327]
OFD	Argon	1700	5	6	5-30 s (in vitro); 10 min (in vivo)	Both	CAP	Phenotype; Mechanism	in vitro (cell)	Proliferation	Solid cancer (breast, bladder)	[46]
OFD	Argon	1100	1.9	6	0.5-3 min	Direct	CAP	Phenotype; Mechanism	in vitro (cell)	Proliferation	Squamous cell cancer	[328]
PDD	Air	10	N/A	8	20 min	Indirect	CAP	Phenotype; Mechanism	in vitro (cell)/in vivo (mice)	Proliferation	Bladder cancer	[329]
DBD	Air	12	N/A	5	1-1.5 min	Direct	CAP	Phenotype	in vitro (skin)	Others	Wound	[15]
OFD	Argon	1000	4	3	0.25-2 min	Both	CAP	Phenotype; Mechanism	in vitro (cell)	Proliferation	Peritoneal Cancer	[330]
PDD	Argon	20.8	3	4.43	0.17-1 min	Both	CAP	Phenotype; Mechanism	in vitro (cell)/in vivo (mice)	Proliferation	Melanoma	[331]
DBD	Argon/Helium	20	5	4.5	1.1-3.58 min	Both	CAP	Phenotype; Mechanism	in vitro (cell)/in vivo (mice)	Proliferation	Breast cancer	[332]
PDD	Air	83	2	1.6	5-20 min	Indirect	CAP	Phenotype; Mechanism	in vitro (cell)	Proliferation	Head & neck cancer	[333]
PDD	Air	50	N/A	N/A	2.5-20 min	Both	CAP combination	Phenotype; Mechanism	in vitro (cell)	Proliferation	Myeloid leukemia	[334]
DBD	Neon	10	N/A	0.22	0.5-2.5 min	Direct	CAP	Phenotype	in vitro (bacteria)	Others	Dentistry	[335]
PDD	Helium	N/A	4	N/A	N/A	Both	CAP combination	Phenotype	in vitro (cell)	Proliferation	Glioblastoma multiforme	[36]
PDD	Air	N/A	N/A	N/A	40 s	Direct	CAP	Phenotype; Mechanism	in vitro (skin)	Degeneration	Pemphigus	[336]
OFD	Air	N/A	N/A	8	90-120 min	Direct	CAP	Phenotype; Mechanism	Others	Others	SARS-CoV-2, IAV, HRV, HAdV, *Pseudomonas aeruginosa*, MRSA	[337]
PDD	Air	N/A	N/A	23	3-15 min	Direct	CAP	Phenotype; Mechanism	in vitro (bacteria)	Others	*Acinetobacter baumannii*	[338]
PDD	Helium	2.14	10.6	N/A	0.5-1 min	Direct	CAP	Phenotype; Mechanism	in vitro (cell)	Proliferation	Breast cancer	[339]
DBD	Air	8	N/A	225	0.5-5 min	Direct	CAP	Phenotype; Mechanism	Others	Others	Environment	[20]
DBD	Air	13560	N/A	4	1-6 min	Direct	CAP	Phenotype; Mechanism	in vitro (cell)	Proliferation	Solid cancer (ovarian, prostate, breast)	[340]
PDD	Helium	13560	6	N/A	1-5 min	Both	CAP	Phenotype; Mechanism	in vitro (bacteria)	Others	*Staphylococcus aureus*	[341]

**Abbreviations:** DBD (Dielectric Barrier Discharge), PDD (Piezoelectric Direct Discharge), OFD (Other Forms of Discharge), CDV (canine distemper virus), CPV (canine parvovirus), CRAB (carbapenem-resistant *Acinetobacter baumannii*), CRPA (Carbapenem-resistant *Pseudomonas aeruginosa*), CRKP (carbapenem-resistant *Klebsiella pneumoniae*), HAdV (Human adenovirus), HRV (Human rhinovirus), MRSA (Methicillin-resistant *staphylococcus aureus*), IAV (Influenza A virus), IBRV (Modified-live infectious bovine rhinotracheitis virus), SARS-CoV-2 (Severe acute respiratory syndrome coronavirus 2).

### 3.1 Frequency

The driving frequency of the applied electric field fundamentally governs the discharge regime, electron kinetics, and subsequent plasma chemistry, thereby shaping the biological efficacy of CAP. CAP devices typically operate across four principal frequency domains: low frequency (e.g., <100 Hz), medium frequency (e.g., 100-10,000 Hz), high frequency (e.g., 10-10,000 kHz), and radio frequency (13.56 MHz, 27.12 MHz, or 40.78 MHz) [342]. Each regime generates distinct RONS signatures, which in turn dictate the resulting biomedical outcomes. It is important to note, however, that similar RONS levels can be achieved across different frequency regimes by adjusting other parameters such as voltage, gas composition, and treatment duration, reflecting the multidimensional nature of parametric control in CAP dosing.

Frequency modulates the number and intensity of microdischarge events per unit time in dielectric barrier discharge (DBD) configurations, one primary type of CAP ejection source. Increasing the driving frequency from 1 kHz to 20 kHz under constant voltage not only elevates average power density and RONS production but also alters gas temperature and discharge uniformity [343]. Comparative studies of identical DBD geometries operated at 50 Hz (low) versus 20 kHz (high) have revealed marked differences in RONS composition. Low-frequency operation favors ozone (O_3_) generation, as extended inter-pulse periods allow for three-body recombination reactions; in contrast, high-frequency operation enhances the formation of nitric oxide (NO) and ONOO^−^, driven by increased electron density and dissociative pathways [[344], [345], [346]]. These chemical disparities translate directly into divergent biological outcomes: low-frequency CAP exhibits enhanced bactericidal activity against Gram-negative pathogens [347], whereas high-frequency CAP demonstrates superior anticancer efficacy in melanoma models [348].

Radio frequency operation at 13.56 MHz is predominantly employed in atmospheric pressure plasma jets for biomedical applications, owing to its efficient power coupling and stable glow discharge at lower voltages, under optimized conditions [349,350]. It is important to note, however, that filamentation behavior is gas-dependent: while helium radio frequency jets typically exhibit diffuse, filament-free operation, argon radio frequency jets can display strong filamentation depending on flow dynamics and operating parameters [351]. The radio frequency cycle time (e.g., 82 ns at 13.56 MHz) is shorter than the characteristic time scales for ion transport [352], resulting in electron-driven chemistry with minimized ion bombardment energy, a feature particularly advantageous for treating sensitive biological substrates such as cells and tissues.

**Fig. 3:**
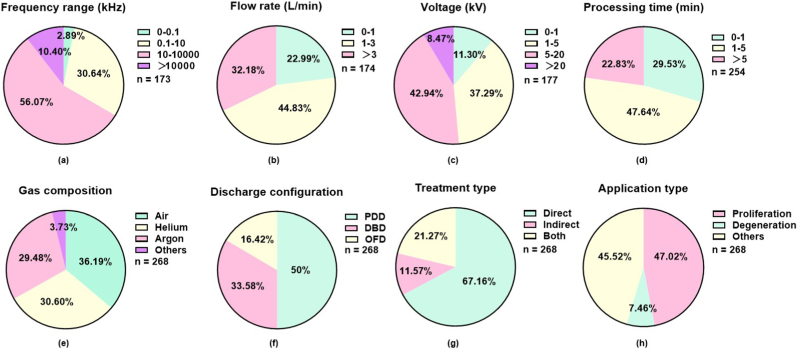
**Quantitative summary of recent (2020**–**2025) CAP studies on therapeutic applications categorized by key operational parameters.** Frequency distribution of studies according to **(a)** frequency range, **(b)** flow rate, **(c)** voltage, **(d)** processing time, **(e)** gas composition, **(f)** discharge configuration, **(g)** treatment type, and **(h)** biomedical application. Studies where CAP was used solely for surface sterilization, disinfection, food processing, or agriculture without therapeutic application were excluded.

A survey of biomedical CAP applications published between 2020 and 2025 reveals a clear predominance of high-frequency plasma ejection, accounting for 60.27% of all studies (Fig. 2a) and 56.07% when focusing specifically on therapeutic applications (Fig. 3a, Table 1), irrespective of source configuration (DBD versus others). This distribution underscores a translational preference for high-frequency operation, likely driven by its capacity to generate a diversified RONS repertoire capable of delivering targeted biological effects across a wide spectrum of therapeutic contexts. The convergence of chemical flexibility, operational stability, and biological efficacy positions high-frequency CAP as the prevailing platform for its clinical translation.

### 3.2 Flow rate

Gas flow rate exerts a dual influence on CAP performance: it determines the residence time of reactive species within the active plasma zone and dictates the convective transport of RONS to the treatment target. Consequently, flow rate optimization requires balancing chemical generation efficiency against delivery efficacy [353,354]. At low flow rates (e.g., 0.25-1.4 slm), extended gas residence time in the discharge region increases per-volume energy deposition and promotes three-body reactions, enhancing the formation of long-lived species such as O_3_ and NO_x_. However, insufficient flow permits ambient air entrainment into the plasma column, diluting the feed gas and introducing uncontrolled RONS [355]. Additionally, low flow rates may result in gas heating, risking thermal damage to biological substrates. At high flow rates (e.g., >3 L/min), convective transport dominates, delivering short-lived species (•OH, •O, ^1^O_2_) to the target before recombination or quenching [356,357]. Yet elevated flow rates can compromise treatment reproducibility, as the fluid dynamics may transition from laminar to turbulent regimes [358]. Furthermore, plasma discharges inherently involve electrohydrodynamic (EHD) phenomena, wherein momentum transfer from charged species to neutral gas molecules in the presence of strong electric fields can induce gas flow modifications, alter the flow regime, and influence the transport of reactive species [[359], [360], [361]]. For instance, computational fluid dynamics simulations indicated that laminar flow (Reynolds number <2000) maintained a concentrated RONS distribution along the jet axis, whereas turbulent mixing dispersed species and reduced the flux delivered to the treatment area [362,363]. Accordingly, most current clinical CAP devices operate in laminar or low-turbulence regimes to ensure reproducible dosing [87,88].

Accordingly, most current clinical CAP devices operate in laminar or low-turbulence regimes to ensure reproducible dosing [364,365]. Thus, to extend CAP efficacy to diseases such as cancer, devices capable of stably producing short-lived species under turbulent conditions are needed. Plasma jet length increases with flow rate up to an optimal value, beyond which turbulence induces jet instability and species dilution. Supporting this need for optimization, Kasih and colleagues systematically varied helium flow rate from 2 to 7 L/min at applied voltages of 1.20-2.00 kV, achieving tunable plasma jet lengths of 5-35 mm while maintaining stable operation at 26 °C [366]. Their findings established that flow rate optimization must occur in conjunction with voltage adjustment, as the voltage required for stable ignition increases with flow rate. Thus, achieving reproducible and clinically effective plasma delivery requires a careful balancing act, i.e., optimizing flow rate in tandem with voltage adjustment to maintain sufficient flux of effective RONS while avoiding the instability and dilution introduced by excessive turbulence, a trade-off that ultimately defines the therapeutic window for CAP in biomedical applications.

During 2020-2025, the majority (42.16%) of studies employed CAP at flow rates of 1-3 L/min, followed by > 3 L/min (36.76%) and <1 L/min (21.08%, Fig. 2b–Table 1); and the 1-3 L/min section further increased to 44.83% when focusing on therapeutics (Fig. 3b). This distribution reflects the combined biological effects of short- and long-lived reactive species. Short-lived radicals react at near diffusion-limited rates over nanometer-to-submicrometer distances, delivering potent, localized actions ideal for triggering rapid cellular responses [367,368]; in contrast, non-radical ROS are more stable and traverse membranes via aquaporins, enabling long-range communication and integrated signaling through structured relay systems [[369], [370], [371], [372], [373]]. While radicals excel in initiating localized bursts to boost mitohormesis or ablate transformed cells, non-radical modalities provide tunable, repeatable signaling for tissue repair and tumor microenvironment preconditioning, properties that can synergize for improved disease management [374]. This explains why 1-3 L/min is the predominant flow rate in most studies especially when focusing on disease treatment, even in the absence of other parameters being uniformly calibrated. Additionally, the cooling effect provided by higher gas flow rates, particularly important for argon-fed devices, which tend to operate at higher gas temperatures than helium systems, helps maintain safe operating temperatures and prevent thermal damage to biological substrates. These factors underscore the unique benefits of applying CAP in addressing complex biomedical conditions like cancers.

### 3.3 Voltage

Applied voltage modulates the Laplacian (background) electric field, which influences the initiation and propagation of ionization waves (streamers) in plasma sources [[375], [376], [377]]. However, the space charge electric field that develops at the streamer head during ionization wave propagation is far more intense than the applied field and constitutes the primary driver of electron energization, RONS generation, and subsequent plasma chemistry [6,359]. Thus, this space charge field may not have a strict direct correlation with the applied voltage, as it depends on complex ionization wave dynamics including gas composition, flow conditions, and electrode geometry. Nevertheless, increasing the peak-to-peak voltage elevates both electron density and mean electron energy, thereby enhancing the quantity and diversity of generated RONS [378], and forming a foundation for dose control in plasma biomedical applications.

In conventional DBD configurations, operation typically employs an alternating current voltage waveform with amplitudes of 2 kV across frequencies of approximately 10-125 kHz [379]. A survey of CAP-related studies reveals distinct voltage preferences: the majority (43.95%) utilized voltages between 5 and 20 kV (excluding 5 kV), followed by 34.08% employing 1-5 kV (excluding 1 kV), while voltages below 1 kV (11.66%) and above 20 kV (10.31%) constituted smaller proportions (Fig. 2c–Table 1). This distribution pattern persisted when analysis was restricted to therapeutic applications, though with a notable shift: a modest increase in studies utilizing the 1-5 kV range, accompanied by a corresponding decrease in higher voltage usage (Fig. 3c). These trends suggest an emerging optimization toward moderate voltage regimes for clinical translation.

The relationship between applied voltage and RONS production is inherently non-linear and regime-dependent. Near the breakdown threshold, discharge operates in filamentary mode characterized by stochastic micro-discharges, with RONS production scaling approximately linearly with excess voltage above ignition [380]. At higher voltages, transition to glow-mode operation achieves uniform discharge with more predictable scaling relationships. Optical emission spectroscopy (OES) demonstrates that •OH and N_2_ emission intensities increase exponentially with applied voltage in helium radio frequency jets, corresponding to enhanced dissociation of ambient water vapor and nitrogen [381]. This exponential relationship underscores the sensitivity of plasma chemistry to voltage modulation.

Voltage modulation enables real-time dose titration with remarkable dynamic range. For instance, increasing voltage from 16 kV to 26 kV in a DBD non-thermal CAP device increased H_2_O_2_ production in treated liquids by approximately ten-fold [382]. Leveraging the hormetic nature of ROS-mediated biological responses, tuning CAP voltage along a gradient may enable controlled transition from cytoprotective effects, such as non-cytotoxic Nrf2 activation suitable for regenerative applications, to cytotoxic effects, including caspase-3-driven apoptosis required for oncotherapy [383]. This voltage-dependent therapeutic window shift exemplifies the parametric control concept: identical device geometry and treatment duration can produce diametrically opposite biological outcomes through voltage adjustment alone, highlighting the critical importance of precise dose calibration.

However, voltage effects cannot be considered in isolation. The actual power delivered to the biological substrate depends on the complete circuit impedance, which varies dynamically with target conductivity, humidity, and proximity [384,385]. Moreover, biological tissues exhibit frequency-dependent dielectric properties that actively load the plasma circuit, rendering voltage-only control insufficient for consistent dose regulation. Tissue hydration, ionic strength, and surface topology collectively influence impedance matching, introducing variability that pure voltage monitoring fails to capture. Consequently, modern CAP systems increasingly incorporate real-time voltage and current probes coupled with impedance matching networks to maintain consistently delivered power despite variable loading conditions [386]. These advancements toward closed-loop control represent critical enabling technology for clinical translation, where reproducible dosing across heterogeneous tissue environments is essential for therapeutic reliability and regulatory approval. Importantly, the implementation of body-mimicking impedance, designing the plasma device to present an impedance that matches that of human tissues, further reduces sensitivity to inter-patient and intra-tissue variability, thereby enhancing treatment reproducibility and facilitating regulatory approval [387].

### 3.4 Processing time

Exposure duration is perhaps the most fundamental and commonly reported dosing parameter in CAP research, as it directly determines the cumulative RONS flux delivered to the biological target. However, its biological impact is modulated by two dynamic processes: the kinetics of RONS accumulation in the treatment medium and the temporal progression of cellular adaptive responses. In direct treatment configurations where plasma impinges directly onto tissue or cell culture, reactive species concentrations at the target increase monotonically with exposure time until reaching a plateau. This equilibrium reflects a balance between ongoing RONS production, chemical decay, and diffusion away from the treatment zone. For typical helium jets treating 1 mL liquid volumes, for instance, H_2_O_2_ accumulates at approximately 3.33 μM/s before approaching steady-state concentration [388].

The biological consequences of extended exposure manifest through cumulative oxidative damage. DNA strand breaks, as quantified by comet assay, exhibit sigmoidal accumulation kinetics characterized by an initial lag phase, a subsequent linear phase corresponding to radical chain reaction propagation, and a final plateau phase indicating saturation [389]. Cell viability follows threshold kinetics: minimal effect is observed until a critical cumulative dose is achieved, followed by exponential decline [70]. This threshold varies substantially between cell types, with cancer cells typically exhibiting lower thresholds than their primary counterparts [81].

Analysis of publications from 2020 to 2025 reveals distinct trends in exposure time selection across biomedical applications. The majority of studies employed treatment durations of 1–5 min (44.59%), followed by < 1 min (27.71%) and >5 min (27.71%), independent of other parameter settings (Fig. 2d–Table 1). When analysis was restricted to therapeutic applications, a preference for shorter processing times emerged: the use of durations under 5 min increased, accompanied by a corresponding decline in longer treatments (Fig. 3d). This trend likely reflects efforts to enhance safety margins in disease treatment by minimizing non-specific tissue damage.

Critically, treatment duration does not act in isolation but interacts synergistically with parameters such as applied voltage and gas composition. Higher voltages or oxygen admixtures can achieve equivalent biological effects in substantially shorter times. Moreover, even when cumulative RONS concentrations are matched, short, high-intensity exposures may elicit different biological outcomes than prolonged low-intensity treatments due to differences in cellular stress response kinetics [390]. This underscores the need for integrated dose metrics that account for both exposure duration and the dynamic composition of reactive species—a critical consideration for standardizing CAP protocols and ensuring reproducible therapeutic outcomes across different device configurations and treatment contexts.

### 3.5 Gas type

Feed gas composition fundamentally determines the RONS repertoire that CAP can generate, rendering gas selection arguably the most influential parameter for therapeutic outcomes. The choice encompasses both the carrier gas (typically helium or argon) and various admixtures, including ambient air, nitrogen, oxygen, and water vapor [391,392]. This parametric decision shapes the entire therapeutic profile, as each gas combination generates a distinct RONS signature with corresponding biological effects.

Analysis of publications from 2020 to 2025 reveals that carrier gas-based CAP is more frequently adopted for therapeutic purposes, accounting for 60.08% of therapeutic studies compared to 56.21% of all CAP-related biological reports (Fig. 2e, **3e**). Helium and argon predominate as carrier gases due to their low breakdown voltage, metastable state-mediated Penning ionization, and chemically inert nature. Helium offers superior discharge stability and lower gas temperature owing to its high thermal conductivity and low molecular weight, which accelerates convective cooling [365,[393], [394], [395]]. Argon provides higher electron density at equivalent power and generates more intense ultraviolet emission, but exhibits greater filamentation tendency and higher gas temperature [396,397]. Over the past five years, slightly more studies employed helium (29.29%) than argon (26.92%) as their carrier gas, despite helium's higher cost (Fig. 2e–Table 1), suggesting that discharge stability and temperature control often outweigh economic considerations in research settings.

Ambient air as feed gas offers compelling practical advantages for clinical translation, including elimination of gas cylinders, enhanced convenience, and reduced cost. Consequently, air ranks first among all gas compositions in studies published between 2020 and 2025 (Fig. 2e–Table 1). However, it is important to recognize that air is not an ideal gas for all applications; rather, its use involves significant trade-offs that must be carefully managed. First, air plasma generates complex RONS mixtures including NOx, ozone, and particulate matter, requiring careful control to avoid potential toxicity and to ensure reproducible biological outcomes [398,399]. Second, the generation of ozone and NOx species necessitates optimization of operating parameters to balance therapeutic efficacy against safety considerations. Third, air-fed plasma devices typically require sufficiently high flow rates to dissipate heat and prevent excessive heat load, as air plasma tends to operate at higher temperatures than noble gas plasmas under comparable conditions. Fourth, air composition varies substantially with ambient humidity, CO_2_ concentration, and trace contaminants, introducing reproducibility challenges that must be addressed through environmental control or real-time monitoring [400]. This trade-off between practical convenience and technical complexity represents a central tension in CAP translational research.

Controlled admixtures enable targeted modulation of plasma chemistry. Oxygen admixture (0.1-5%) dramatically increases production of atomic oxygen, singlet oxygen, and ozone while reducing nitrogen-derived species. Oxygen-enriched CAP demonstrates enhanced bactericidal activity [401,402] and anticancer efficacy, but carries increased risk of excessive oxidative damage to normal tissue. Nitrogen admixture promotes peroxynitrite and nitric oxide generation, favoring immunomodulatory and vasodilatory applications [403]. Water vapor admixture, introduced via gas humidification or derived from ambient humidity, generates •OH radicals through electron impact dissociation and Penning reactions with metastable helium [404]. These distinct chemical profiles enable rational selection of gas composition based on target pathology and desired biological mechanism.

Recent developments in gas blending strategies have advanced beyond fixed compositions toward programmable RONS generation. Combinatorial plasma processing systems can continuously vary gas ratios during treatment, creating libraries of plasma conditions on single substrates for high-throughput optimization [405,406]. Such systems represent the frontier of parametric control, transitioning gas composition from a static parameter to a dynamic dosing variable capable of real-time adjustment. This emerging capability holds particular promise for combination therapies requiring sequential exposure to different RONS environments, for example, initial immunomodulatory nitrogen-based treatment followed by cytotoxic oxygen-based dosing. As these technologies mature, the conceptual framework for gas composition will continue evolving from simple carrier selection toward sophisticated, temporally modulated plasma chemistry engineering.

## 4 Dual-index integrative framework for CAP dosage

**Fig. 4:**
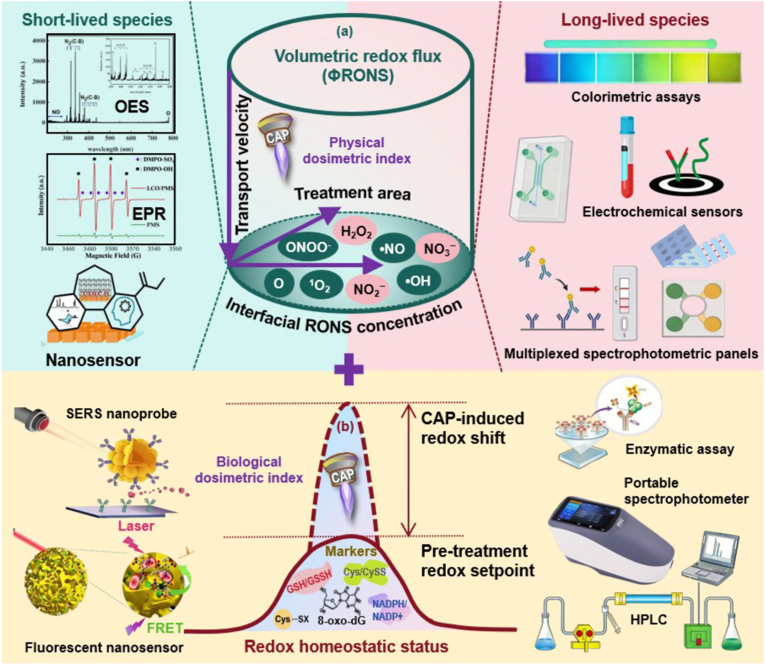
**Dual-index integrative framework for cold atmospheric plasma dosage.** CAP devices generate RONS through adjustable operating parameters including frequency, flow rate, voltage, processing time, and gas composition, which collectively determine the quantity, composition, and transport of RONS delivered to the biological target. **(a)** Volumetric redox flux (ΦRONS) as the physical dosimetric index. ΦRONS integrates three determinant factors: interfacial RONS concentration ([RONS]_i_), transport velocity (V_transport_), and treatment area (A_treatment_). Operationally, ΦRONS is quantified via accumulated long-lived RONS (H_2_O_2_, NO_2_^−^, NO_3_^−^) in standardized liquid targets using colorimetric/fluorometric assays, electrochemical sensors, or multiplexed spectrophotometric panels. Short-lived species (•OH, ^1^O_2_, O, •NO, ONOO^−^) are assessed via OES, EPR, or emerging nanosensors, serving as mechanistic correlates and real-time proxies. **(b)** Redox homeostatic status as the biological dosimetric index. Redox homeostatic status captures the biological impact through endogenous markers reflecting the pre-treatment redox setpoint and CAP-induced shifts. Candidate markers include glutathione (GSH/GSSG), cysteine/cystine (Cys/CySS), NAD(P)H/NAD(P)^+^ ratios, protein thiol oxidation (peroxiredoxins, KEAP1), and oxidative damage products (8-oxo-dG, 4-HNE, malondialdehyde). Detection platforms range from enzymatic assays and HPLC to electrochemical sensors, SERS nanoprobes, fluorescent nanosensors, and portable spectrophotometers. **Abbreviations:** CAP, cold atmospheric plasma; RONS, reactive oxygen and nitrogen species; ΦRONS, volumetric redox flux; DBD, dielectric barrier discharge; PDD, piezoelectric direct discharge; OES, optical emission spectroscopy; EPR, electron paramagnetic resonance; HPLC, high performance liquid chromatography; SERS, surface-enhanced Raman scattering; FRET, fluorescence resonance energy transfer; SERS, surface-enhanced raman spectroscopy; HPLC, high-performance liquid chromatography; GSH, reduced glutathione; GSSG, oxidized glutathione; Cys, cysteine; CySS, cystine; 4-HNE, 4-hydroxynonenal; 8-oxo-dG, 8-oxo-7,8-dihydroguanosine; KEAP1, Kelch-like ECH-associated protein 1; Nrf2, nuclear factor erythroid 2-related factor 2.

The preceding sections establish that CAP dose is multidimensional, comprising distinct parametric inputs that collectively determine biological outcome. The central challenge for clinical translation is consolidating this complexity into quantifiable, reproducible, and biologically relevant dose metrics. We propose a dual-index integrative framework that addresses both the physical delivery of reactive species and the biological response they evoke. This framework comprises: (i) volumetric redox flux as the physical dosimetric index governing parametric control, and (ii) redox homeostatic status as the biological dosimetric index reflecting cell heterogeneity and therapeutic sensitivity (Fig. 4). Together, these twin indices bridge the gap between plasma device operation and clinical efficacy, enabling rational dose specification and adaptive treatment optimization.

### 4.1 Volumetric redox flux: parametric control of CAP dosage

Volumetric redox flux (ΦRONS) is defined as the total flux of all RONS delivered to the target surface per unit area per unit time. It consolidates three determinant factors: (1) the concentration of individual RONS species at the plasma-liquid or plasma-tissue interface ([RONS]ᵢ), (2) the transport velocity of reactive species to the target (V_transport_), and (3) the effective treatment area (A_treatment_). As a physical metric, ΦRONS serves as the mediating variable that translates adjustable operating parameters, i.e., voltage, frequency, gas composition, flow rate, processing time, into quantifiable dose delivery.

#### 4.1.1 Theoretical basis

In principle, the dosimetric concept ΦRONS encompasses both long-lived species such as H_2_O_2_, NO_2_^−^, NO_3_^−^, ONOO^−^-derived products and short-lived radicals including •OH, ^1^O_2_, O, •NO. However, the sub-millisecond to nanosecond half-lives of short-lived radicals preclude their direct *ex situ* quantification under ambient conditions. For practical implementation, therefore, ΦRONS is operationally defined via the accumulated concentrations of long-lived RONS, principally H_2_O_2_, NO_2_^−^, NO_3_^−^, in standardized liquid targets under defined treatment geometries [43,407].

This pragmatic reduction to long-lived species can be justified on four interconnected grounds. First, long-lived species exhibit sufficient chemical stability, with half-lives ranging from hours to days, enabling reliable *ex situ* quantification. This contrasts sharply with •OH (half-life nanoseconds), which decays before conventional measurement techniques can capture it [408]. Second, H_2_O_2_ concentrations correlate strongly with cytotoxic outcomes across multiple cell types and CAP devices, with IC_50_ values typically ranging from 20 to 300 μM [409]. This quantitative relationship provides a direct bridge between plasma chemistry and biological effect. Third, the relative ratio of H_2_O_2_ to NO_2_^−^/NO_3_^−^ predicts the balance between oxidative and nitrosative stress, thereby influencing downstream signaling pathways, for instance, steering cellular responses toward cytoprotective Nrf2 activation versus apoptotic commitment [410,411]. Fourth, long-lived RONS accumulate linearly with exposure time under constant operating conditions, enabling straightforward dose interpolation and protocol scaling [381].

Crucially, long-lived species function as dosimetric surrogates that integrate the production and delivery history of their short-lived progenitors. The concentration of H_2_O_2_ measured post-exposure reflects the cumulative influence of •OH and O radicals that underwent recombination or dismutation; similarly, NO_2_^−^ and NO_3_^−^ arise from the decay of •NO, •NO_2_, and ONOO^−^ [412]. Thus, while ΦRONS as operationally defined does not directly quantify short-lived radicals, it embeds their integrated contribution within a measurable framework.

It is important to note, however, that this dosimetric framework is complicated by the presence of biological targets. In cell-free systems, the gas/liquid phase chemical balance can be reliably characterized, as short-lived species either recombine, react with liquid-phase components, or contribute to long-lived species formation. However, in the presence of cells or tissues, short-lived species are rapidly consumed through interactions with cellular membranes, proteins, and other biological targets. This biological consumption not only drives the primary biological effects, including membrane permeabilization, protein oxidation, and signaling pathway activation, but also alters the subsequent gas/liquid phase chemical balance, reducing the contribution of short-lived species to long-lived product formation. Consequently, measurements of long-lived species in cell-free systems may not fully reflect the RONS flux experienced by biological targets under treatment conditions, and ΦRONS should be understood as a dosimetric surrogate that provides a reproducible, device-agnostic metric for comparative dose specification rather than a complete measure of biological exposure.

Despite the practical centrality of long-lived species, short-lived RONS cannot be dismissed from the theoretical framework. Radicals such as •OH, atomic oxygen, and singlet oxygen are the primary agents of immediate oxidative damage to membranes, proteins, and nucleic acids; they also initiate the chemical cascades that yield H_2_O_2_ and nitrite. Peroxynitrite (ONOO^−^), although short-lived, mediates both cytotoxic nitrative stress and anti-inflammatory signaling. Consequently, any comprehensive dosimetric framework must account for short-lived species, either through direct measurement or via validated correlations with their long-lived products.

ΦRONS therefore exists on a conceptual continuum. The operational definition based on long-lived species serves routine dose specification, quality control, and inter-laboratory comparability, essential functions for translational progress. Meanwhile, the theoretical construct encompassing all RONS informs mechanistic understanding and advanced process control, guiding the rational design of plasma conditions for targeted therapeutic applications. This dual perspective acknowledges both the practical constraints of clinical implementation and the mechanistic depth required for scientific rigor, positioning ΦRONS as a unifying dosimetric concept that bridges fundamental plasma chemistry with biomedical application.

#### 4.1.2 Methodologies

Quantification of ΦRONS employs a tiered methodological approach tailored to the intended application and required temporal resolution, ranging from routine *ex situ* measurements to real-time *in situ* monitoring and theranostic integration.

First, chemical probe-based quantification constitutes the gold standard for long-lived RONS analysis. Colorimetric and fluorometric assays remain the reference methods: H_2_O_2_ can be routinely measured via horseradish peroxidase-catalyzed oxidation of Amplex Red or Amplex UltraRed or via titanium oxysulfate spectrophotometry [413,414]. Nitrite may be quantified by the Griess reaction, while nitrate requires prior reduction to nitrite via nitrate reductase or vanadium(III) chloride [415,416]. Recent advances have enabled multiplexed spectrophotometric panels that simultaneously quantify H_2_O_2_, NO_2_^−^, and NO_3_^−^ from single 100 μL samples within 2 min [417,418]. These assays are performed on standardized liquid targets, typically phosphate-buffered saline, cell culture medium, or deionized water, with defined volume (e.g., 1-5 mL) and surface area (e.g., 1-10 cm^2^) to ensure geometric reproducibility across experiments and laboratories [419].

Second, electrochemical sensing represents an emerging paradigm for real-time dose monitoring. Electrochemical sensor arrays functionalized with Prussian Blue, hemin, or carbon nanotubes enable amperometric detection of H_2_O_2_ and NO_2_^−^ with sub-second response times [420,421]. Recent prototypes integrate multiple working electrodes on single chips [[422], [423], [424]], potentially enabling multiplexed RONS quantification directly in the treatment liquid during plasma exposure. These systems require initial calibration against standard assays but offer the critical advantage of online dose feedback, facilitating closed-loop control of plasma delivery [425].

Third, short-lived species diagnostics address the need to characterize the transient radicals that initiate biological effects but evade conventional quantification. Optical emission spectroscopy (OES) provides semi-quantitative, real-time proxies for short-lived radical densities [426]. Although absolute calibration by actinometry or broadband absorption spectroscopy remains challenging under atmospheric conditions, relative emission intensities correlate reliably with downstream H_2_O_2_ yields and biological outcomes. Electron paramagnetic resonance (EPR) spectroscopy with spin traps enables *ex vivo* quantification of specific radical species in liquids or tissue homogenates, though temporal resolution is limited to minutes [427]. Fluorescent nanosensors incorporating boronate-caged dyes or lanthanide probes are under development for *in situ* detection of •OH at bio-interfaces, promising to bridge the gap between plasma physics and biology [428].

Fourth, long-lived species theranostics translate dosimetric quantification into predictive and therapeutic tools. Multi-laboratory validation studies have established that volumetric flux of long-lived species such as H_2_O_2_ and NO/O_2_ can help predict biological responses. For instance, NO/O_2_ flux was positively associated with the expression of angiogenesis markers including BDNF, TrkB, VEGF, and tubulin [429]. This concept is exemplified by two complementary strategies: a dual-locked NIR probe (MB-*m*-borate) designed for precise melanoma detection via H_2_O_2_-tyrosinase cascade-activated methylene blue (MB) release, and H_2_O_2_-responsive protein biomimetic nanoparticles (MnO_2_-ICG@BSA) developed for melanoma therapy [430]. These approaches exemplify how ΦRONS quantification can extend beyond dosimetry to enable image-guided therapy and personalized treatment planning.

Collectively, this tiered methodological framework accommodates the full spectrum of CAP dosimetry needs, from routine quality control using chemical probes, through real-time process monitoring via electrochemical sensors, to mechanistic investigation of short-lived radicals and theranostic integration. The selection among these methods depends on the specific research or clinical objective, the required temporal resolution, and the balance between measurement precision and practical implementability. As CAP technology advances toward clinical translation, the convergence of multiple quantification modalities will be essential for establishing robust dose-response relationships and ensuring reproducible therapeutic outcomes.

#### 4.1.3 Challenges

The translation of ΦRONS from a conceptual dosimetric framework into routine clinical practice necessitates addressing four fundamental challenges that span measurement methodology, biological complexity, and technological integration. These challenges define the frontier of CAP dosimetry research and must be systematically resolved to enable reproducible therapeutic outcomes.

First, short-lived species integration. The operational reliance on long-lived surrogates, while pragmatically necessary, introduces systematic uncertainty when short-lived radicals dominate the biological effect. Conditions that enhance •OH production, such as humidified feed gas or high-voltage operation, may yield greater cytotoxicity than predicted from H_2_O_2_ concentration alone. Conversely, NO-rich plasmas may exert anti-inflammatory effects not captured by NO_2_^−^/NO_3_^−^ accumulation. Bridging this gap requires either: (i) development of robust, low-cost sensors for real-time short-lived species quantification capable of operating in the challenging plasma environment, or (ii) establishment of validated transfer functions linking optical emission spectroscopy (OES) ratios to long-lived yields across device classes and operating envelopes. Such transfer functions would enable inference of short-lived radical influence from routinely measurable long-lived concentrations, preserving practical utility while enhancing mechanistic fidelity.

Second, RONS synergism. The dosimetric framework must account for RONS interactions, not merely individual concentrations. Combined exposure to H_2_O_2_ and NO_2_^−^ produces synergistic cytotoxicity through peroxynitrite formation, either pre-formed in the plasma-activated liquid or generated intracellularly from co-diffusing species [431,432]. Short-lived species amplify this synergy: •OH initiates lipid peroxidation, creating chain reactions that propagate oxidative damage long after plasma exposure ceases. Volumetric redox flux therefore requires characterization of the complete RONS profile, including concentration ratios among long-lived species and, ideally, estimates of short-lived radical influence. Single-parameter surrogates (e.g., H_2_O_2_ alone) prove insufficient for applications where the H_2_O_2_:NO_2_^−^ ratio varies substantially with operating conditions, as occurs when switching between nitrogen-rich and oxygen-rich feed gas compositions [433]. This interdependence necessitates multidimensional dose specification that captures both absolute concentrations and relative ratios.

Third, protocol standardization. Implementation of volumetric redox flux as a standard dosimetric requires consensus on measurement protocols across the rapidly expanding CAP research community. Critical variables demanding harmonization include: treatment liquid composition (water, saline, buffer, or cell culture medium), liquid volume-to-surface area ratio, time interval between treatment and measurement, temperature during storage and analysis, and specific quantification methodology. Small variations in these parameters substantially alter RONS accumulation rates [[434], [435], [436], [437]], rendering inter-laboratory comparison invalid without strict standardization. The International Society for Plasma Medicine has initiated working groups to establish these standards, paralleling historical developments in radiation dosimetry that enabled quantitative radiotherapy. Separate guidelines for short-lived species diagnostics—specifying OES calibration targets, optical collection geometry, spin trap concentrations, and temporal acquisition windows—are similarly being drafted to ensure cross-laboratory comparability. These standardization efforts may represent essential infrastructure for the current translational progress.

Fourth, real-time control integration. Current dosimetric approaches remain predominantly retrospective, measuring RONS accumulation in collected samples post-treatment. This temporal disconnect precludes adaptive dose adjustment during the therapeutic session itself. While OES provides real-time emission data, the correlation between emission intensity and delivered redox flux to tissue surfaces remains semi-quantitative and device-specific. Fully closed-loop control, wherein measured dose automatically adjusts operating parameters (voltage, frequency, gas composition, exposure duration) to maintain a specified RONS delivery setpoint, awaits establishment and clinical validation. Achieving this capability requires integration of fast-response electrochemical sensors or calibrated OES systems with intelligent control algorithms capable of compensating for variable tissue properties, humidity, and proximity effects. Such systems represent the ultimate expression of precision plasma medicine, wherein dose delivery is actively maintained rather than passively recorded.

Collectively, these four challenges may define the roadmap for CAP dosimetry research. Their systematic resolution, through sensor development, mechanistic understanding, community standardization, and control integration, may transform ΦRONS from a descriptive metric into a prescriptive tool enabling reproducible, personalized plasma therapeutics.

### 4.2 Redox homeostatic status: biological index of CAP dosage

Volumetric redox flux quantifies what is delivered to the target; it does not, however, capture what the target experiences. Identical redox flux can induce cytoprotection or death depending on the cell type, its metabolic state, antioxidant capacity, and microenvironment. This biological variability necessitates a complementary dosimetric index that reports the cellular response to CAP exposure. We propose redox homeostatic status as the integrative biological index of CAP dosage.

#### 4.2.1 Theoretical basis

CAP exerts its therapeutic effects through precise modulation of cellular redox homeostasis [58]. The unifying principle governing these effects is hormesis: low-to-moderate oxidative challenges activate adaptive antioxidant responses, while high-intensity challenges overwhelm cellular defense mechanisms and trigger death pathways [54]. Critically, the therapeutic window for a given redox flux is not fixed but is instead determined by the pre-treatment redox setpoint of the target cell population. Cells existing in a pro-oxidant state, including many cancer cells, inflamed tissues, and ischemic myocardium, exhibit heightened sensitivity to further oxidative challenge, whereas cells with replete antioxidant reserves (such as quiescent fibroblasts or resting neurons) tolerate substantially higher redox fluxes before exhibiting cytotoxicity.

This biphasic, setpoint-dependent response provides the theoretical foundation for proposing redox homeostatic status as a biological dosimetric index. From this perspective, the goal of CAP therapy is not merely to deliver a prescribed redox flux, but to deliberately shift cellular redox state from a pathological setpoint toward a homeostatic optimum. For hyper-oxidative pathologies, including cancers and inflammatory skin diseases, CAP must elevate oxidative stress beyond the death threshold, pushing cells past a point of no return. For hypo-oxidative conditions such as neurodegenerative disorders, CAP must activate endogenous antioxidant machinery to restore redox balance and support cellular survival and regeneration.

Thus, CAP dosing can be reformulated as a closed-loop control problem with two interconnected state variables: the physical delivered dose (ΦRONS) and the biological response state (redox homeostatic status). The controller adjusts ΦRONS based on real-time or near-real-time assessment of cellular redox status, titrating the physical dose to achieve and maintain the desired biological setpoint. This conceptual framework may help transform CAP from a fixed-protocol intervention into an adaptive, feedback-driven therapeutic modality, one that acknowledges biological variability and actively compensates for it, rather than treating all targets with identical physical parameters. Realizing this vision requires integrated development of rapid redox biosensors, validated biomarkers of setpoint status, and intelligent plasma delivery systems capable of dose adjustment on therapeutic timescales.

#### 4.2.2 Methodologies

Implementation of redox homeostatic status as a biological dosimetric index necessitates two interdependent prerequisites: the identification of feasible markers or marker panels that reliably report cellular redox levels under diverse pathological conditions, and the establishment of detection technologies capable of quantifying these markers with sufficient speed, sensitivity, and practicality for clinical decision-making. Meeting these requirements is essential for transitioning CAP from empirical dosing to biologically guided therapy.

First, candidate redox markers. Multiple classes of endogenous molecules collectively reflect the complex landscape of cellular redox balance. GSH and its oxidized disulfide form (GSSG) constitute the principal thiol redox buffer of the cell; the GSH/GSSG ratio typically decreases from normal reducing conditions to oxidized states associated with oxidative stress and pathology [438]. The cysteine/cystine (Cys/CySS) couple couples extracellular redox state to intracellular signaling and transport processes. Pyridine nucleotide ratios, NAD^+^/NADH and NADP^+^/NADPH, report metabolic redox status, anabolic capacity, and the reducing power available for antioxidant regeneration [439]. Protein thiol oxidation, particularly of redox-sensitive proteins such as peroxiredoxins, thioredoxin, and KEAP1, provides proximal readouts of specific signaling pathway activation, capturing early events in the cellular stress response [440]. Lipid peroxidation products, including 4-hydroxynonenal and malondialdehyde, and DNA oxidation products such as 8-oxo-2′-deoxyguanosine (8-oxo-dG) indicate macromolecular oxidative damage with established links to disease pathogenesis and therapeutic response [441].

For clinical applications, marker selection must balance mechanistic relevance with analytical tractability. GSH/GSSG in peripheral blood mononuclear cells or tissue biopsies can be reliably quantified by enzymatic recycling assays or high-performance liquid chromatography; however, sample processing requires rapid acidification to prevent artefactual oxidation that would invalidate clinical interpretation [442,443]. Extracellular H_2_O_2_ efflux from treated tissues, measurable by microdialysis or amperometric sensors positioned near the treatment site, correlates with intracellular peroxide tone and offers near-real-time monitoring capability [444]. Real-time imaging of genetically encoded redox-sensitive fluorescent proteins (roGFP, HyPer, Grx1-roGFP) may enable dynamic monitoring with subcellular resolution in experimental systems but faces substantial translational barriers for clinical application due to delivery and safety considerations [445].

It is worth emphasizing that no single redox marker universally reports therapeutic response across all pathologies. The redox pathology of atherosclerosis differs fundamentally from that of neurodegeneration or cancer, and the temporal dynamics of marker changes vary with treatment intensity and cellular context. A tiered approach to marker implementation is therefore needed: (i) disease-specific marker panels reflecting the dominant redox pathology of the target condition, e.g., lipid peroxidation products for atherosclerosis, protein carbonyls for neurodegeneration, GSH depletion for cancer, may provide diagnostic context and baseline characterization; (ii) treatment-responsive markers that change rapidly following CAP exposure, such as Nrf2 target gene expression or extracellular H_2_O_2_ efflux, may enable early assessment of biological effect and dose titration during therapy; and (iii) outcome-predictive markers validated against clinical endpoints, for example, 8-oxo-dG reduction correlating with tumor response or GSH recovery predicting wound healing, may support treatment optimization and prognostic assessment.

Second, emerging detection platforms. Translating redox markers into clinically actionable information requires detection technologies that balance analytical performance with practical implementability. Electrochemical sensors functionalized with thiol-reactive mercury or silver interfaces enable voltammetric quantification of GSH and cysteine in microliter sample volumes within seconds, offering the speed necessary for intraprocedural decision-making. These sensors can be miniaturized and integrated into handheld devices for point-of-care deployment. Surface-enhanced Raman spectroscopy (SERS) nanoprobes bearing maleimide or boronate moieties permit multiplexed detection of multiple thiol species and reactive oxygen-nitrogen species simultaneously, providing comprehensive redox profiling from single small-volume samples. Fluorescent nanosensors incorporating redox-responsive dyes such as resazurin or coumarin boronate into biocompatible hydrogels or microneedle arrays are under active development for transdermal redox monitoring, potentially enabling non-invasive or minimally invasive assessment of treatment effects in superficial tissues targeted by CAP. Portable spectrophotometers adapted for clinical use may quantify urinary 8-oxo-dG and F_2_-isoprostanes as systemic oxidative stress markers [446,447], offering a practical approach for longitudinal monitoring of patient redox status across multiple treatment sessions.

The convergence of marker biology with detection technology creates a pathway for implementing redox homeostatic status as a practical clinical tool. Optimal implementation will likely combine a core panel of mechanistically informative markers, GSH/GSSG ratio for overall redox buffer status, extracellular H_2_O_2_ for real-time dose monitoring, and a disease-relevant damage marker, with technology platforms selected for the specific clinical context, i.e., electrochemical sensors for intraprocedural guidance, SERS for comprehensive profiling, and portable spectrophotometry for longitudinal follow-up. This integrated approach may transform redox homeostatic status from a theoretical concept into an actionable dosimetric index, enabling biologically guided CAP therapy that accounts for individual patient variability and dynamic treatment response.

#### 4.2.3 Challenges

The translation of redox homeostatic status from a theoretical dosimetric index into a clinically actionable biomarker requires systematic resolution of five interconnected challenges spanning measurement science, biological variability, and technological integration. These challenges may define the research frontier for personalized plasma medicine and must be addressed to enable real-time, adaptive dosing.

First, marker specificity and causality. Redox markers currently provide correlative rather than causal information about biological outcomes. Elevated 8-oxo-dG confirms oxidative DNA damage but does not distinguish lethal genotoxicity from repairable insult. Increased GSH may indicate successful Nrf2-mediated adaptation or a futile compensatory response preceding cell death. Distinguishing adaptive from pathological redox changes requires kinetic context: transient GSH elevation followed by normalization indicates successful hormetic adaptation, whereas persistent GSH depletion signals impending cell death. Establishing causal relationships between specific marker dynamics and definitive biological endpoints such as survival, death, senescence may thus be essential for interpreting marker data in therapeutic decision-making.

Second, inter-individual variability. Basal redox status varies substantially with age, nutritional status, medication use, and comorbid conditions. A GSH/GSSG ratio considered normal for a healthy young adult may indicate profound depletion in an elderly diabetic patient with compromised antioxidant reserves. Similarly, baseline 8-oxo-dG levels differ between individuals based on metabolic rate, smoking history, and environmental exposures. Establishing normative ranges for candidate redox markers across demographic strata and pathological states is a prerequisite for clinical deployment. Without such reference data, it remains impossible to determine whether a measured value represents a pathological state requiring intervention or a normal variant requiring none.

Third, temporal dynamics. Redox status is not static but fluctuates with circadian rhythm, metabolic state, and ongoing inflammation. Single time-point measurements may misrepresent the integrated cellular response to CAP treatment. Optimal implementation of redox homeostatic status as a dosimetric index requires understanding of the characteristic response kinetics for each marker, e.g., minutes to hours for Nrf2 target gene expression, and hours to days for GSH recovery, as well as specification of standardized sampling windows relative to treatment. This temporal framework enables interpretation of whether a measured value reflects peak response, recovery phase, or steady-state adaptation.

Fourth, tissue accessibility. Deep tissues like brain, myocardium and visceral organs, are not amenable to direct redox probe application or biopsy during routine CAP therapy. Surrogate matrices including plasma, urine, and exhaled breath condensate may provide systemic but not tissue-specific redox information, potentially missing localized effects at the treatment site. Microdialysis probes inserted into accessible tissues (subcutaneous, muscle) enable local sampling but are invasive and impractical for routine clinical use. Non-invasive imaging modalities such as electron paramagnetic resonance imaging and hyperpolarized ^13^C magnetic resonance spectroscopy offer promise for tissue-specific redox assessment but remain confined to specialized research centers, far from routine clinical implementation.

Fifth, real-time dose monitoring and closed-loop control. Current dosimetric approaches remain predominantly retrospective, measuring RONS accumulation in collected samples post-treatment. This temporal disconnect precludes adaptive dose adjustment during the therapeutic session itself. Emerging solutions leverage OES feedback, where specific emission lines—•OH at 309 nm, N_2_ at 337 nm, O_2_ at 777 nm—serve as real-time proxies for RONS production [448]. Machine learning algorithms trained on optical spectra can now predict H_2_O_2_ yields with predictive accuracy (R^2^) exceeding 0.998 [449], demonstrating the feasibility of software-based dose estimation from plasma emissions. Fully closed-loop control, wherein measured dose automatically adjusts operating parameters to maintain a specified RONS delivery setpoint, has been demonstrated in prototype systems using model predictive control. However, integration of biological state variables (redox homeostatic status) into the control loop remains experimental [450], representing the ultimate frontier: a system that not only maintains physical dose constancy but actively titrates that dose based on real-time assessment of the patient's redox response.

Collectively, these five challenges delineate a comprehensive research agenda. Their systematic resolution—through marker validation, normative database development, kinetic characterization, accessible measurement technologies, and biologically integrated closed-loop control—may eventually transform redox homeostatic status from a conceptual index into a practical tool guiding personalized plasma therapeutics.

## 5 Limitations and future direction

Despite significant progress in parametric control and dual-index dosimetric quantification, multiple barriers impede the routine clinical translation of CAP therapy.

### 5.1 Device heterogeneity

A fundamental source of technical heterogeneity in CAP medicine lies in the discharge configuration. For the purpose of quantitative analysis, we categorized devices based on their underlying discharge mechanism. The most prevalent categories in the 2020–2025 literature were: piezoelectric direct discharge (PDD), dielectric barrier discharge (DBD), including both planar and jet-type DBD configurations, and other forms of discharge (OFD), encompassing devices operating on distinct principles like radio frequency capacitively coupled jets (e.g., kINPen), corona discharge and microwave discharge. Analysis of publications reveals that PDD accounts for 47.63% of studies, DBD-based devices accounts for 38.46%, and OFD accounts for 13.91% (Fig. 2f–Table 1). When the analysis is restricted to therapeutic applications specifically, the share of PDD further raises to 50% after excluding other biological applications (Fig. 3f). This distribution reflects the translational momentum of PDD platforms, likely driven by their operational simplicity and focused delivery characteristics.

DBD and PDD systems embody fundamentally different physical principles of plasma generation. DBD systems generate plasma through microdischarges between electrodes separated by one or more insulating dielectric layers, enabling large-area treatment under ambient air without requiring noble gas feed [314,315,451,452]. Commercial exemplars such as the PlasmaDerm VU-2010 demonstrate the clinical viability of this approach for dermatological indications [453]. PDD systems, in contrast, exploit the resonant vibration of piezoelectric transformers to produce high electric fields that directly ionize the surrounding gas, typically yielding a focused, jet-like plasma plume without requiring external high-voltage power supplies [454,455]. The Adtec SteriPlas represents a widely adopted commercial PDD platform, particularly in wound healing applications [456]. Beyond this fundamental discharge dichotomy, CAP devices diverge further across multiple engineering dimensions: electrode geometry, power coupling efficiency, driving frequency bandwidth, and feed gas delivery modality. These differences collectively render direct parameter transfer between systems scientifically invalid. A treatment protocol meticulously optimized for the kINPen MED, a well-characterized argon plasma jet (categorized to OFD), cannot be meaningfully applied to the PlasmaDerm VU-2010 DBD device or to a PDD-based platform with distinct electrical and fluidic characteristics. This device-specific parameter dependence has historically hindered cross-laboratory reproducibility and slowed translational progress. Though ΦRONS may offer a promising device-agnostic dosimetric metric that transcends these configurational differences, yet standardized protocols for ΦRONS measurement, encompassing liquid composition, volume-to-area ratio, post-treatment timing, and multiplexed quantification, remain under active development and validation across device classes. The establishment of such standards is thus a prerequisite for realizing the full potential of dose-based rather than parameter-based protocol specification.

Addressing device heterogeneity requires a multi-pronged strategic approach that moves beyond parameter harmonization toward fundamental dosimetric standardization. First, adoption of ΦRONS as a universal reporting metric must be mandated across all CAP device classes. Investigators should report, at minimum, the accumulated H_2_O_2_, NO_2_^−^, and NO_3_^−^ concentrations in a standardized reference liquid—for example, phosphate-buffered saline with defined volume (e.g., 1 mL) and surface area (e.g., 2 cm^2^)—under fixed treatment geometry, alongside traditional operating parameters. This practice may help enable cross-device comparison and meta-analysis even when underlying discharge mechanisms differ fundamentally, creating a common language for dose specification. Second, establishment of reference transfer functions between device classes is urgently needed to enable protocol translation. For each major device category (DBD, PDD, OFD), a consensus reference device should be designated and characterized exhaustively across its operating envelope. Secondary devices can then be calibrated against this reference by establishing empirical correlations between their respective ΦRONS outputs under matched biological endpoints. Such calibration campaigns require coordinated multi-laboratory efforts; the International Society for Plasma Medicine and the COST Action CA20114 (Plasma applications for smart and sustainable agriculture) have initiated analogous frameworks that may be extended to medical devices, providing institutional infrastructure for dosimetric harmonization. Third, development of open-access repositories for device-specific dosimetric data would accelerate knowledge transfer and protocol optimization. A centralized database documenting ΦRONS yields, RONS composition ratios, and associated biological efficacy endpoints for diverse devices under standardized measurement conditions would enable machine learning-assisted parameter extrapolation and reduce redundant optimization efforts across laboratories. Such repositories may represent a community resource with compounding value as more devices are characterized. Fourth, evolution of regulatory pathways to recognize device-agnostic dosimetry is essential for clinical translation. Rather than requiring identical operating parameters for clinical protocol approval, an approach that locks innovation to specific legacy devices, regulators may want to accept validated ΦRONS delivery ranges with demonstrated biological equivalence across devices. This paradigm shift, from parameter-based to dose-based approval, parallels the historical transition in radiation oncology that enabled diverse treatment platforms to deliver standardized therapeutic doses. Implementing this framework would substantially lower the translational barrier for new CAP devices entering the clinic while maintaining rigorous safety and efficacy standards. Collectively, these four strategic elements may constitute a roadmap for transforming CAP from a collection of device-specific empirical practices into a coherent therapeutic modality with transferable dosimetry. The convergence of universal reporting, reference calibration, open data infrastructure, and dose-based regulation may enable the field to transcend current heterogeneity and deliver reproducible, personalized plasma therapeutics across the full spectrum of redox-modulated diseases.

### 5.2 Treatment form diversity

The clinical translation of CAP is further complicated by the expanding repertoire of application modalities that have emerged over the past five years (Fig. 2g and. 3g, Table 1). Beyond conventional direct tissue exposure, wherein the plasma plume impinges directly onto the target surface, CAP can now be delivered indirectly via plasma-activated liquids (PALs) and plasma-functionalized hydrogels. While these indirect formats offer substantial practical advantages in terms of accessibility, storage stability, and off-the-shelf deployment, they introduce a fundamental dosimetric challenge: the componentry of CAP preserved across different application forms is neither identical nor directly intercomparable, and no consensus currently exists on how to calibrate these diverse formulations into a uniform, device-agnostic dose matrix.

Direct CAP application exposes the biological target to the full repertoire of plasma components, including the complete spectrum of short-lived radicals, long-lived species, electric fields, ultraviolet radiation, and charged particles [457]. This multimodal delivery enables immediate interactions with cell membranes and rapid initiation of signaling cascades. Indirect applications, by contrast, involve pre-treating a liquid medium or hydrogel carrier with CAP, followed by subsequent administration of the activated vehicle to the biological target. PALs, typically saline, buffer solutions, cell culture media, or Ringer's lactate [[458], [459], [460], [461]] stabilize long-lived species generated during plasma exposure, while short-lived radicals decay during or immediately after the activation process [462,463]. The resulting RONS profile is therefore fundamentally different from direct CAP, comprising only those species with sufficient half-life to survive the activation-to-administration interval. Plasma-activated hydrogels, fabricated by incorporating plasma-treated solutions into polymer networks or by direct plasma treatment of gel precursors, offer tunable RONS release kinetics and topical applicability [462,464], but introduce additional complexity through polymer-RONS interactions that can sequester, quench, or gradually release reactive species.

Despite the expanding interest in indirect approaches, direct treatment continues to dominate the field, accounting for 70.12% of all CAP studies published over the past five years (Fig. 2g–Table 1). This predominance reflects both historical precedent and the intuitive appeal of direct plasma-tissue interaction. However, when analysis is restricted specifically to therapeutic applications, a modest but informative shift emerges: the combined use of direct and indirect modalities increased from 17.75% in the general dataset (Fig. 2g) to 21.27% in therapeutic-focused studies (Fig. 3g). This rise highlights the expanding therapeutic landscape and underscores the imperative for context-dependent adaptation of conventional CAP protocols. Different clinical scenarios—wound healing, cancer treatment, dermatological conditions—may preferentially benefit from one modality over another, necessitating a flexible yet standardized approach to dose specification.

The fundamental dosimetric challenge resides in the divergent RONS profiles preserved across application forms. Direct CAP delivers a complex, time-varying RONS cocktail dominated by short-lived radicals at the moment of exposure. PALs retain only long-lived species, with H_2_O_2_:NO_2_^−^:NO_3_^−^ ratios determined by the gas composition, activation time, liquid chemistry, and post-treatment storage conditions [3,43,[465], [466], [467]]. Hydrogels introduce additional variables: polymer matrix interactions can sequester, quench, or gradually release RONS, and the effective diffusivity of each species within the gel differs by orders of magnitude depending on mesh size, charge, and crosslinking density [[468], [469], [470]]. Critically, the biological effects of these modalities are not equivalent even when normalized to H_2_O_2_ concentration, the most commonly reported dosimetric for PALs. PALs often exhibit enhanced cytotoxicity compared to direct CAP at equivalent H_2_O_2_ doses [471], attributable to synergistic H_2_O_2_/NO_2_^−^ chemistry that generates secondary reactive species and to the absence of short-lived radical-mediated cellular repair activation [472]. Conversely, direct CAP may prove superior for applications requiring immediate membrane interactions or rapid electrical field effects, where short-lived radicals and physical plasma components contribute substantively [473]. Thus, H_2_O_2_ concentration alone is an insufficient surrogate for cross-modality dose comparison, and more sophisticated dosimetric frameworks are urgently required.

Establishing dose equivalence across direct and indirect CAP modalities necessitates a multi-tiered calibration framework that acknowledges both the commonalities and distinctions among application forms. First, common currency metrics must be defined and validated across modalities. The ΦRONS index, based on accumulated concentrations of H_2_O_2_, NO_2_^−^, and NO_3_^−^, provides a device-agnostic physical dosimetric for PALs and hydrogels, analogous to its established role in direct CAP dosimetry. However, because indirect formats lack short-lived radical contributions, ΦRONS targets must be adjusted to reflect the absence of these effectors. This necessitates empirical determination of ‘biological equivalence factors’ that relate PAL H_2_O_2_:NO_2_^−^ ratios and absolute concentrations to direct CAP outcomes for specific therapeutic applications. Such factors would enable clinicians to prescribe, for example, ‘a PAL with ΦRONS equivalent to 3 min of direct CAP exposure for wound healing’, providing a common language for dose specification across modalities. Second, standardized PAL and hydrogel preparation protocols must be established through consensus processes. Critical variables requiring harmonization include: activation liquid composition (buffering capacity, amino acid content, ionic strength, presence of scavengers), liquid volume-to-surface area ratio during activation, activation duration and plasma source characteristics, post-treatment storage temperature and duration, and for hydrogels, polymer type, crosslinking density, loading method, and release kinetics. The International Society for Plasma Medicine has recognized this gap and is currently developing technical specifications for PAL production and quality control [467]. These standards will enable reproducible manufacturing and cross-laboratory comparability, prerequisites for regulatory approval and clinical deployment. Third, predictive correlations between preparation parameters and biological activity must be developed using advanced analytical methods. Machine learning models trained on multidimensional datasets—encompassing activation parameters, liquid composition, storage conditions, RONS profiles, and biological endpoints—can identify optimal formulation windows and enable quality-by-design approaches to PAL and hydrogel manufacturing [474]. Such models would transform indirect CAP applications from empirically optimized formulations, which require extensive trial-and-error optimization for each new condition, to rationally designed, reproducibly manufactured therapeutic products with predictable biological activity. This capability is essential for scaling production and enabling multi-center clinical trials. Fourth, modality-specific biological reference points must be established for key therapeutic indications. For wound healing, this might involve calibration against standardized *ex vivo* skin models with defined healing metrics. For oncology, panels of representative cancer cell lines with characterized redox sensitivity could serve as biological dosimeters. These reference points would enable cross-modality comparison of biological potency independent of physical RONS measurements, providing an orthogonal validation framework for dose equivalence claims. Fifth, regulatory pathways must evolve to accommodate modality-specific dosimetry. Rather than requiring identical physical dose metrics across application forms, an approach that ignores fundamental differences in RONS delivery, regulators should accept modality-appropriate dose specification backed by validated biological equivalence data. A PAL formulated to deliver a defined ΦRONS range with characterized H_2_O_2_:NO_2_^−^ ratio should be approvable based on demonstrated biological equivalence to direct CAP for a specific indication, even though the physical delivery mechanisms differ. This flexible yet rigorous approach would accelerate innovation while maintaining patient safety.

In summary, the diversification of CAP application forms, while clinically advantageous, has outpaced the development of cross-modality dosimetric standards. Direct and indirect CAP are not interchangeable delivery vehicles but complementary modalities with distinct RONS signatures, biological optima, and clinical niches. Direct CAP remains essential for applications requiring immediate short-lived radical effects and multimodal plasma-tissue interaction. PALs offer practical advantages for off-the-shelf deployment and treatment of internal or hard-to-reach anatomical sites. Hydrogels provide tunable release kinetics ideal for topical and sustained delivery applications. A unified calibration matrix, grounded in common currency RONS metrics, informed by machine learning-optimized formulation strategies, and validated against modality-specific biological reference points, is urgently needed to enable regulatory approval and clinical deployment of the full spectrum of plasma-activated therapeutics. This framework will transform CAP from a single-modality intervention into a versatile therapeutic platform capable of addressing diverse clinical scenarios with reproducible, personalized dosing.

## 6 Conclusion

The clinical translation of CAP has been constrained not by a lack of therapeutic efficacy—indeed, a growing body of evidence confirms its biological activity across diverse disease models—but by the absence of a coherent, universally accepted framework for dose specification and delivery. Through systematic delineation of the dose-dependent biology of CAP and the critical parameters that modulate its biological effects, we arrive at a central thesis: CAP dose is neither a single operating parameter (voltage, frequency, exposure time) nor a single measurable output (H_2_O_2_ concentration), but a multidimensional construct requiring dual-index quantification that integrates both physical delivery and biological response.

The hormetic, biphasic nature of CAP biological effects provides the foundational principle for dose stratification. In general, moderate-to-high redox fluxes induces cell demise via overwhelming cellular antioxidant capacity or improving oxygenation and perfusion [475,476], serving applications in proliferative syndromes such as oncology; low-to-moderate fluxes activate Nrf2 signaling, elevate glutathione, and confer cytoprotection, offering therapeutic potential for neurodegenerative disorders, chronic inflammatory conditions, and other degenerative pathologies where oxidative stress constitutes a core pathogenic mechanism. Critically, the transition threshold between these opposing outcomes is not fixed but shifts dynamically with cell type, metabolic state, redox setpoint, and microenvironmental context, a biological variability that physical dosimetry alone cannot resolve. Analysis of publications from 2020 to 2025 reveals that considerably less attention has been devoted to CAP applications in degenerative syndromes, which account for only 6.21% of all studies (Fig. 2h–Table 1). When analysis was restricted to therapeutic applications specifically, this figure increased only modestly to 7.46% (Fig. 3h), a substantially smaller rise compared to that observed for proliferative diseases, which surged from 37.28% to 47.02% (Fig. 3h). This disparity likely reflects a limited understanding of CAP's dual biological effects and the absence of established dosage guidelines for degenerative conditions, highlighting a significant yet underexplored avenue for future investigation that warrants coordinated research effort.

In response to these challenges, we have proposed a dual-index integrative framework that couples physical and biological dose metrics into a unified dosing architecture. The ΦRONS index, operationally defined via accumulated concentrations of H_2_O_2_, NO_2_^−^, and NO_3_^−^ in standardized liquid targets under defined treatment geometries, serves as the mediating variable that translates adjustable operating parameters, frequency, flow rate, voltage, processing time, gas composition, and delivery modality, into quantifiable dose delivery. Extensive validation across multiple device classes and biological models demonstrates that ΦRONS predicts biological outcomes with superior precision to any single operating parameter, capturing the integrated effect of complex plasma chemistry in a single dosimetric quantity. However, ΦRONS reports only what is delivered to the biological target, not what is experienced by the target cells. As highlighted in recent work on plasma penetration [75], short-lived radicals and physical plasma components are largely confined to superficial layers while long-lived RONS may reach deeper structures, a stratification of species that ΦRONS alone may not capture. Redox homeostatic status, assessed through context-specific panels of reduced and oxidized glutathione (GSH/GSSG), cysteine/cystine ratio, NAD(P)H autofluorescence, and oxidative damage markers such as 8-oxo-dG and lipid peroxidation products, provides the complementary biological dosimetric index that captures the cellular response and defines the individual therapeutic window for each patient and tissue context.

This dual-index framework fundamentally reframes CAP dosing as a closed-loop control problem with two interconnected state variables. The outer control loop regulates biological outcome via a redox status setpoint determined by the clinical objective, i.e., cytoprotection for degenerative conditions, cytotoxicity for proliferative diseases. The inner loop maintains the prescribed ΦRONS through real-time parametric adjustment of plasma operating conditions. Realizing this vision requires addressing the dual challenges imposed by device heterogeneity and treatment form diversity, which demands a unified, multi-tiered strategy centered on dosimetric standardization rather than parameter harmonization.

Central to this strategy is the mandatory adoption of ΦRONS, quantified via accumulated concentrations of long-lived species in standardized reference liquids, as a universal reporting metric across all CAP device classes and application modalities. For device heterogeneity, this necessitates the designation of consensus reference devices for each major discharge configuration, i.e., DBD, PDD and OFD, against which secondary devices are calibrated through empirically derived transfer functions linking ΦRONS outputs to matched biological endpoints. Such calibration campaigns require coordinated multi-laboratory collaboration and the establishment of open-access repositories like CAPmed-BC [261] which would archive device-specific dosimetric data, RONS composition ratios, and associated biological outcomes to enable meta-analysis and machine learning-assisted parameter extrapolation.

For treatment form diversity, ΦRONS serves as a common currency physical dosimetric for PALs and hydrogels, but must be supplemented by biological equivalence factors that account for the absence of short-lived radical contributions in indirect formats. This requires the development of standardized protocols for PAL and hydrogel preparation, encompassing activation parameters (gas composition, treatment duration, plasma source characteristics), liquid composition (buffering capacity, amino acid content, ionic strength), post-treatment storage conditions (temperature, duration, light exposure), and matrix properties for hydrogels (polymer type, crosslinking density, loading method). These protocols must be complemented by machine learning-driven predictive models that correlate multidimensional formulation variables with RONS profiles and biological outcomes, enabling quality-by-design approaches to manufacturing and reducing the empirical optimization burden that currently hinders translational progress.

Together, these measures may establish a device-agnostic, cross-modality dosimetric framework that enables dose-based regulatory approval, protocol transferability between laboratories and devices, and the rational design of reproducibly manufactured plasma-activated therapeutics. This framework may transform CAP from a collection of device-specific empirical practices into a coherent therapeutic modality with transferable dosimetry, addressing the fundamental barriers that have historically constrained clinical translation.

The trajectory of parametric control in plasma medicine parallels the historical evolution of radiation oncology, a discipline that underwent a analogous transformation in the mid-twentieth century. Early radiotherapy prescribed dose as exposure time, ‘treat for 5 min’, with corresponding irreproducibility, unpredictable toxicity, and inability to compare outcomes across treatment centers. The adoption of absorbed dose as a standardized metric, enabled by ionization chamber dosimetry and later by image-guided adaptive delivery, transformed radiation therapy into a quantitatively precise, personally optimized discipline capable of delivering curative doses to tumors while sparing adjacent normal tissues. The emergence of RONS dosimetry, real-time optical emission spectroscopy feedback, electrochemical sensor arrays, and machine learning-assisted control algorithms positions CAP to undergo a similar transformation: from an empirical tool of variable reproducibility to a precisely titratable therapeutic modality governed by quantitative, device-agnostic, and biologically interpretable dose metrics.

The parametric control framework presented here does not diminish the complexity of plasma-biological interactions. That is, parameters remain interactive, RONS cocktails remain compositionally rich, and cellular responses remain context-dependent that are influenced by genetic, epigenetic, and environmental factors. Yet these challenges are tractable when approached through structured dosimetric architecture that acknowledges complexity while providing practical tools for dose specification. By embracing ΦRONS as the physical index and redox homeostatic status as the biological index of CAP dosage, the field may accelerate the translational pathway from laboratory discovery to reproducible, personalized, and clinically indispensable therapy. The dose, at last, may be prescribed, not merely administered, a distinction that marks the maturation of CAP from experimental technology to precision therapeutic platform.

## 7 Methods

### 7.1 Search strategy and study selection

This review synthesized evidence on the dose-dependent biological effects, parametric control, and dosimetric frameworks for CAP in biomedical applications. A systematic literature search was conducted in PubMed/MEDLINE, Web of Science, and Scopus databases for articles published between January 2020 and December 2025. The search was restricted to this five-year window and English-language publications to capture the most recent advances in parametric control and dosimetric standardization, reflecting the rapid evolution of plasma medicine research globally.

The search terms included combinations of: ‘cold atmospheric plasma’, ‘cold atmospheric plasmas’, ‘cold atmospheric-pressure plasma’, ‘non-thermal plasma’, ‘nonthermal plasma’, ‘CAP’, ‘dose’, ‘dosimetry’, ‘dosing’, ‘dosimetric’, ‘parameter’, ‘frequency’, ‘voltage’, ‘flow rate’, ‘gas composition’, ‘treatment time’, ‘reactive oxygen species’, ‘ROS’, ‘reactive nitrogen species’, ‘RNS’, ‘redox’, ‘oxidative stress’, ‘cytotoxicity’, ‘cytoprotection’, ‘cancer’, ‘autoimmune’, ‘degeneration’, ‘wound healing’, ‘neuroprotection’, ‘inflammation’, ‘plasma activated liquid’, ‘plasma activated medium’, and ‘hydrogel’.

Inclusion criteria were: (1) original research articles, systematic reviews, and meta-analyses; (2) studies reporting quantitative CAP operating parameters (frequency, voltage, flow rate, gas composition, treatment time); (3) studies quantifying RONS production or reporting dose-response relationships; (4) studies evaluating biomedical outcomes (cell viability, proliferation, apoptosis, oxidative stress markers, redox signaling) following CAP treatment; (5) studies addressing CAP applications in oncology, autoimmune diseases, degenerative disorders, wound healing, neuroprotection, or inflammatory conditions; (6) studies with clearly defined experimental conditions and reproducible methodologies.

Exclusion criteria were: (1) case reports, conference abstracts, editorials, commentaries, and unpublished preprints; (2) studies using plasma temperatures exceeding 50 °C; (3) studies focusing exclusively on plasma physics, engineering, or material processing without biological outcome reporting; (4) studies with insufficient methodological detail to extract parametric data or biological outcomes.

Equivalent search strategies were adapted for Web of Science and Scopus. Additional studies were identified through manual screening of reference lists from included articles and relevant systematic reviews.

### 7.2 Data extraction and parameter categorization

Titles and abstracts retrieved from the database searches were screened against the eligibility criteria. Full texts of potentially relevant articles were obtained and assessed for final inclusion. Data were extracted from included studies using a standardized form that captured:

CAP device characteristics: Discharge configuration (dielectric barrier discharge, DBD; piezoelectric direct discharge, PDD; other forms of discharge, OFD), electrode geometry, power coupling method.

Operating parameters: Frequency (categorized as <100 Hz, 100-10000 Hz, 10-10000 kHz, radio frequency); flow rate (categorized as <1 L/min, 1-3 L/min, >3 L/min); voltage (categorized as <1 kV, 1-5 kV, 5-20 kV, >20 kV); processing time (categorized as <1 min, 1-5 min, >5 min); gas composition (carrier gas: helium, argon, air; admixtures: oxygen, nitrogen, water vapor).

Treatment modality: Direct CAP ejection, plasma-activated liquids or hydrogels.

Biological model: Cell type, animal model, *ex vivo* tissue, or human subjects.

Biological outcomes: Cell viability/proliferation/apoptosis markers, oxidative stress markers, redox signaling.

### 7.3 Quantitative synthesis and parametric mapping

Extracted parametric data were aggregated to generate the quantitative distributions presented in Fig. 2 and Table 1. For each parameter (frequency, flow rate, voltage, processing time, gas composition, discharge configuration, treatment form, and application area), studies were categorized based on reported values or qualitative descriptions. Percentages were calculated relative to the total number of studies reporting each parameter. Studies reporting multiple parameter conditions or multiple applications were counted in all applicable categories.

For application areas, studies were classified into four categories based on the primary therapeutic focus (1) proliferative diseases (e.g., oncology, hyperproliferative disorders): [36,46,320,322,323,325,[327], [328], [329], [330], [331], [332], [333], [334],^339^,^340^] (2) degenerative diseases (e.g., neurodegeneration, discdegeneration); [312,313,319,321,324,336]; (3) others (including wound healing and tissue repair [114,242]; inflammatory conditions like dermatitis and periodontitis [318,335]; infection like bacterial or viral infection [316,317,326,337,338,341]). Classification was based on stated study objectives and biological endpoints.

### 7.4 Evidence grading and framework development

The retrieved literature was evaluated using a modified Oxford Centre for Evidence-Based Medicine (OCEBM) levels of evidence framework adapted for parametric and mechanistic studies. For dose-response relationships and parametric effects, studies with systematic parameter variation and quantitative RONS measurement were prioritized. For biological mechanism elucidation, studies employing genetic or pharmacological interventions were considered higher quality for establishing causality.

The dual-index integrative framework (volumetric redox flux and redox homeostatic status) was developed through iterative synthesis of the extracted evidence according to the following methodology:

Identification of mediating variables: All studies reporting correlations between operating parameters and biological outcomes were reviewed to identify candidate mediating variables. The five parameters identified to compose volumetric redox flux (ΦRONS) were found most frequently and consistently reported physical metrics based on studies under condiseration.

Biological response mapping: Studies reporting differential cellular response to CAP were analyzed to establish the relationship between cellular redox status and biological outcome in response to the same external oxidative perturbation. This analysis informed the need of selecting appropriate redox markers for the biological dosimetric index.

### 7.5 Limitations and methodological considerations

Several methodological limitations inherent to this synthesis approach are acknowledged. First, the heterogeneity of CAP devices, operating parameters, and reporting standards across studies may introduce inconsistency in parametric categorization. Where parameter values were not explicitly reported, studies were excluded from quantitative parametric mapping. Second, the majority of biological evidence derives from in vitro and in vivo models, with variable translation to clinical applications. Third, the five-year search window (2020-2025) captures recent advances but may exclude foundational studies that established parametric relationships; however, key foundational references were retained through citation tracking and manual addition where essential for mechanistic context. Fourth, the proposed dual-index framework represents a conceptual synthesis rather than a quantitative meta-analysis, and prospective validation studies across multiple device classes and applications are required to establish its clinical utility. Where evidence was conflicting or insufficient, this is explicitly noted in the text, and areas requiring further investigation are highlighted in the future directions.

## Funding

Scientific Research Project of Henan Zhongyuan Medical Science and Technology Innovation and Development Foundation (No. ZYYC2023ZD), Youth Science Award Project of the Provincial-level Joint Fund for Science and Technology Research and Development Project in Henan Province (No. 225200810084).

## CRediT authorship contribution statement

**Xiaofeng Dai:** Conceptualization, Data curation, Formal analysis, Investigation, Project administration, Visualization, Writing – original draft, Writing – review & editing. **Jitian Li:** Funding acquisition. **Tanzeela Nawaz:** Data curation. **Chaoqun Ding:** Data curation.

## Declaration of competing interest

The authors declare no conflict of interest.

## Data Availability

The authors do not have permission to share data.
